# Orthogonal Tri‐Modular Coiled‐Coil Assembly for Programmable Multi‐Cargo Display on *Escherichia coli* Nissle 1917

**DOI:** 10.1002/smll.202512642

**Published:** 2026-03-20

**Authors:** Yong Joon Cho, Gyu‐Jin Lee, Jong‐Ha Park, Sung In Lim

**Affiliations:** ^1^ Department of Chemical Engineering Pukyong National University Busan Republic of Korea

**Keywords:** coiled‐coil interactions, live biotherapeutic products, multiplex surface display, orthogonal assembly, programmable living scaffold

## Abstract

Live biotherapeutic products (LBPs) represent emerging living medicines capable of site‐specific intervention; however, current strategies for surface functionalization rely predominantly on static genetic fusion or covalent modification, limiting multiplexing and dynamic reconfiguration. Here, we engineer TriSCs (Tri‐specific Scaffold Cells), a tri‐modular and orthogonally programmable living scaffold based on *Escherichia coli* Nissle 1917. Three distinct α‐helical motifs are displayed on the bacterial outer membrane, forming a reconfigurable biointerface that enables spontaneous, highly specific, and reversible payload assembly via coiled‐coil interactions. This mix‐and‐go strategy allows simultaneous and selective recruitment of multiple functional modules without structural interference. Spatial co‐display of a DR5 agonistic nanobody and an EGFR‐targeting nanobody produces enhanced anticancer activity compared to soluble combinations, underscoring the impact of surface‐organized signaling. In parallel, modular incorporation of a fluorescent reporter enables real‐time bioimaging. By integrating orthogonality, reversibility, and multiplex capability within a single living chassis, TriSCs establish a dynamic and programmable LBP platform for precision theranostics and next‐generation bioengineered therapeutics.

## Graphical abstract

A tri‐specific scaffold cell (TriSC) platform is engineered to enable programmable, reversible, and multiplex surface assembly through orthogonal and spontaneous coiled‐coil interactions. Independent targeting and therapeutic nanobodies are modularly recruited onto bacterial surfaces, allowing tunable combinatorial functionality. This system demonstrates controllable payload exchange and synergistic anticancer activity, offering a versatile strategy for multifunctional live biotherapeutics.

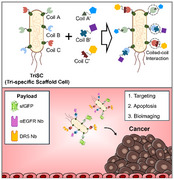

## 1 Introduction

Live biotherapeutic products (LBPs) are engineered microorganisms administered for therapeutic or diagnostic purposes [1, 2]. Their ability to colonize nutrient‐rich or hypoxic microenvironments has attracted considerable interest for treating cancer, metabolic disorders, and inflammatory diseases [3, 4]. Among available microbial chassis, *Escherichia coli* Nissle 1917 (EcN) stands out for its non‐pathogenic profile, lack of virulence factors, and immunomodulatory properties [5, 6, 7, 8]. Its long‐standing clinical use as the probiotic Mutaflor confirms safety, while its well‐characterized genome enables versatile engineering for a wide range of biomedical applications through recombinant technology [8, 9, 10, 11]. A notable example is SYNB1891, an EcN strain that produces a STING agonist, which has advanced into clinical trials, demonstrating both the feasibility and safety of recombinant LBPs in oncology [12, 13, 14]. Another example is SYNB1934, an engineered EcN strain expressing phenylalanine ammonia lyase with enhanced catalytic activity and demonstrated biosafety [15, 16]. To maximize therapeutic potential, strategies that expand the functional versatility and targeting precision of LBPs are essential.

Surface engineering has emerged as a promising approach, allowing localization of cytokines, antibodies, or cytotoxic agents directly on the bacterial membrane. Such modifications can enhance targeting specificity, pharmacokinetics, and therapeutic efficacy [17, 18, 19, 20, 21]. For instance, non‐pathogenic *E. coli* K‐12 DH5α engineered to display a decoy‐resistant IL‐18 mutein elicited strong antitumor responses [19, 21], and EcN microbots carrying doxorubicin and gold nanorods improved tumor penetration and combined chemo‐photothermal therapy [22]. These examples illustrate the potential of surface functionalization but also highlight critical limitations of existing approaches. Direct genetic fusion to membrane anchors often causes folding burden and loss of functionality [21, 23, 24, 25, 26, 27]. Modular coupling strategies such as SpyTag‐SpyCatcher or biotin‐streptavidin alleviate this burden but create irreversible and binary interactions, which limit precise tunability [25, 28, 29, 30, 31, 32]. Moreover, their relatively large size and heterologous origin can impose folding constraints on fused payloads and raise concerns of immunogenicity. Oligonucleotide hybridization offers reversibility but requires chemical modification and is highly susceptible to nuclease degradation [33, 34].

**FIGURE 1:**
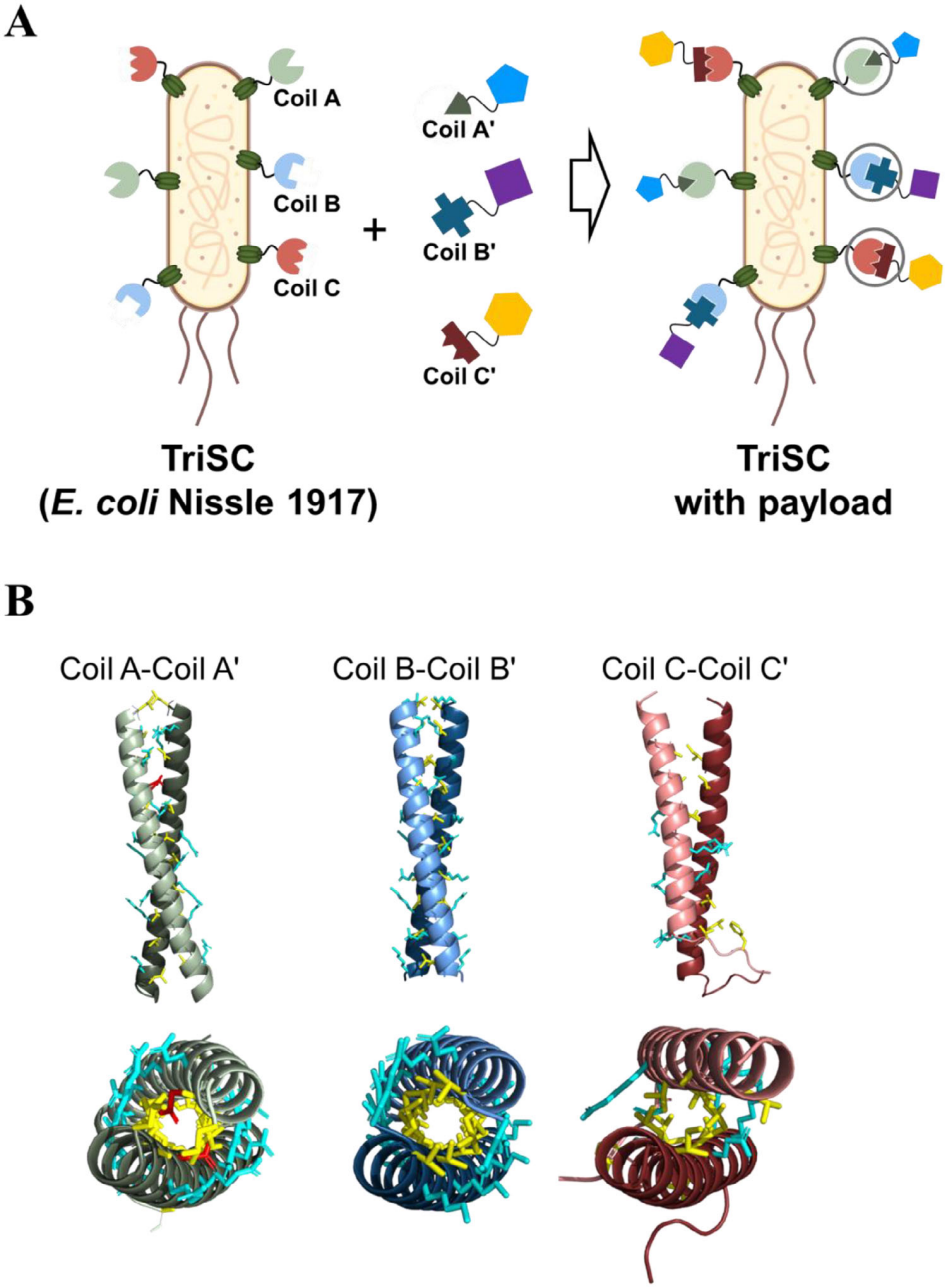
Schematic representation of TriSCs. (A) Modular and orthogonal surface decoration of TriSCs through specific molecular interaction between membrane‐anchored α‐helical domains and their respective partner domains fused to desired payloads. (B) Key residues of the coiled‐coil structures. Hydrophobic residues, ionic/polar residues, and asparagine lock are highlighted in yellow, cyan, and red, respectively.

Here, we present a modular and orthogonally programmable platform based on EcN engineered to display three distinct α‐helical domains via the Lpp–OmpA membrane anchor, termed TriSCs (Tri‐specific Scaffold Cells, Figure 1A). Each α‐helical domain and its complementary α‐helix specifically associate to form a coiled coil through sequence‐encoded hydrophobic and electrostatic interactions (Figure 1B), enabling high‐affinity yet reversible pairing [35, 36, 37, 38, 39, 40, 41, 42, 43]. By integrating the tumor‐tropism of EcN with the coiled‐coil platform, TriSCs provide a multifunctional scaffold for tunable and multiplexed surface functionalization, with potential applications in drug delivery and diagnostics.

## 2 Results and Discussion

### 2.1 Design Principles of a Tri‐Specific Display System

**FIGURE 2:**
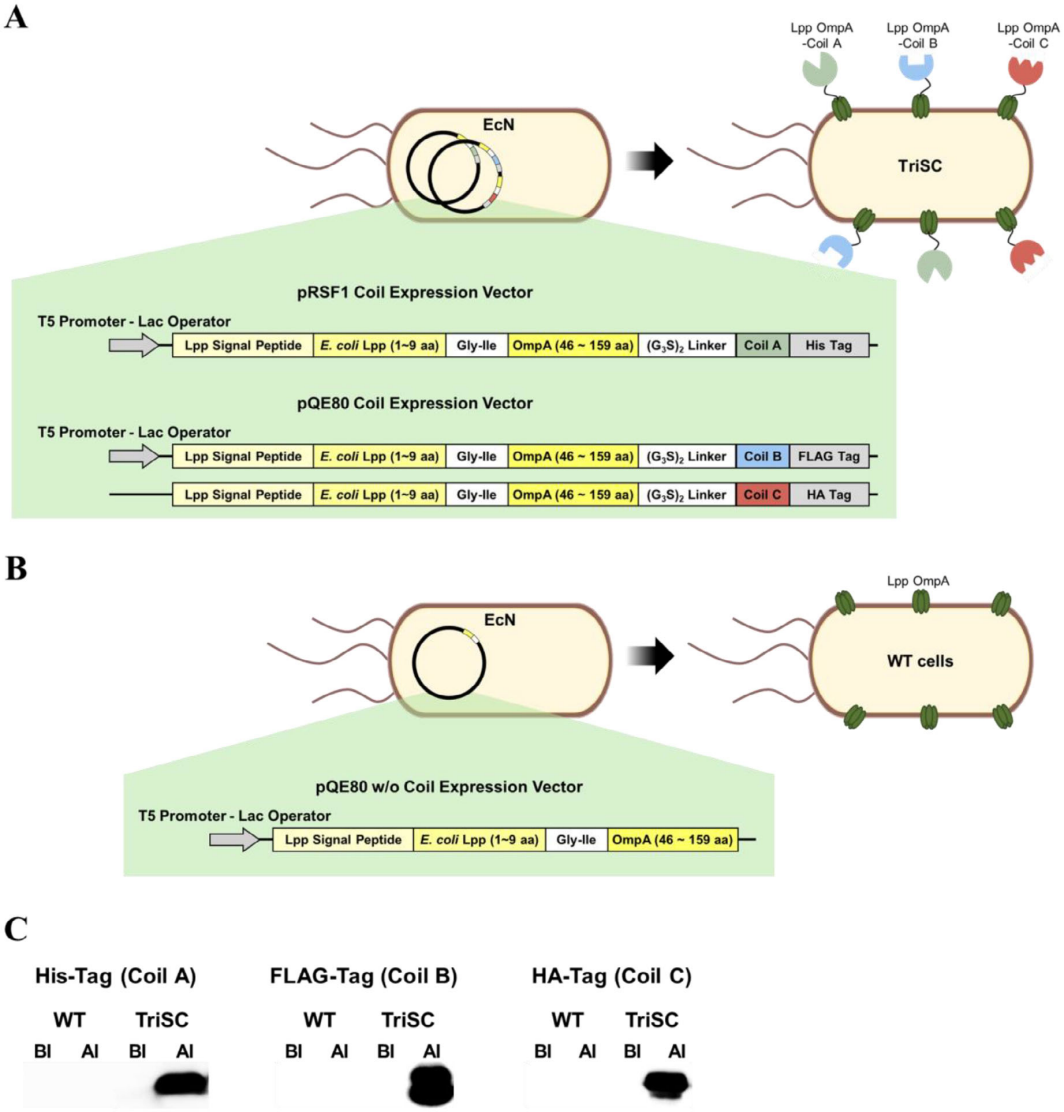
Construction and validation of three coil motifs in EcN. (A) Schematic representation of TriSCs harboring plasmids encoding Lpp–OmpA fused to coil motifs. (B) Schematic representation of WT cells harboring a plasmid encoding Lpp–OmpA without any coil motif. The pRSF1 Coil vector encodes Lpp–OmpA fused to Coil A with a C‐terminal His tag. The pQE80 Coil vector encodes two Lpp–OmpA fusions containing Coil B and Coil C tagged with FLAG and HA, respectively. The pQE80 w/o Coil vector encodes Lpp–OmpA only. (C) Western blot analyses using tag‐specific antibodies. BI: cell lysate collected before IPTG induction; AI: cell lysate collected after IPTG induction.

To enable programmable surface assembly, we employed the Lpp–OmpA fusion as the anchoring domain for EcN. Lpp–OmpA is a compact and well‐characterized scaffold that has been widely validated for surface display of heterologous proteins and supports efficient localization with minimal folding burden [26, 44, 45, 46]. Three coiled‐coil pairs were then selected according to the following criteria (Table S3): formation of orthogonal heterodimers ensuring selective binding without cross‐reactivity; high binding affinity to enable robust surface assembly; and compact size to minimize interference with membrane localization and host metabolic burden. First, Coil A‐Coil A′ was chosen for its complementary charge distribution at critical heptad positions. Coil A contains multiple glutamic acid residues at the e and g positions, together with a positively charged arginine at the g position in the fifth heptad, while Coil A′ provides the inverse arrangement (Figure S1A) [47, 48]. This pattern promotes directional electrostatic pairing. Both helices also contain asparagine in the second heptad that acts as an asparagine lock, further stabilizing heterodimer formation [41, 47]. Coil B‐Coil B′ was chosen as a highly regular synthetic zipper. Its stability derives from repetitive arrays of glutamic acid or lysine at e and g positions, generating extended salt‐bridge ladders (Figure S1B) [49]. The hydrophobic interface is formed from almost exclusively valine and leucine at a and d positions, producing a tightly interlocked core that favors selective and robust dimerization [49, 50, 51]. Coil C‐Coil C′ was selected to introduce a natural, orientation‐distinct interaction mode. Unlike the parallel zippers, this pair forms an antiparallel coiled‐coil. The reversed geometry alters charge alignment, such that electrostatic complementarity arises predominantly between e‐e or g‐g positions rather than e‐g (Figure S1C) [52]. Additional ionic and hydrogen‐bonding contacts, together with a patchwork of hydrophobic residues, generate an asymmetric stabilization pattern [39, 53]. These features minimize potential cross‐reactivity with parallel motifs, thereby enhancing system‐wide orthogonality. EcN was transformed with genes encoding Coil A, B, and C, each individually fused to the C‐terminus of Lpp–OmpA with a distinct C‐terminal epitope tag: Coil A with a His tag, Coil B with a FLAG tag, and Coil C with an HA tag, yielding TriSCs (Figure 2A). EcN expressing only the Lpp–OmpA anchor served as WT cells (Figure 2B). Western blotting analysis of cell lysates using tag‐specific antibodies confirmed robust expression of the three coil motifs specifically in TriSCs (Figure 2C; Figure S2).

### 2.2 Dose‐Dependent Assembly and Specificity of Single‐Payload Interactions

**FIGURE 3:**
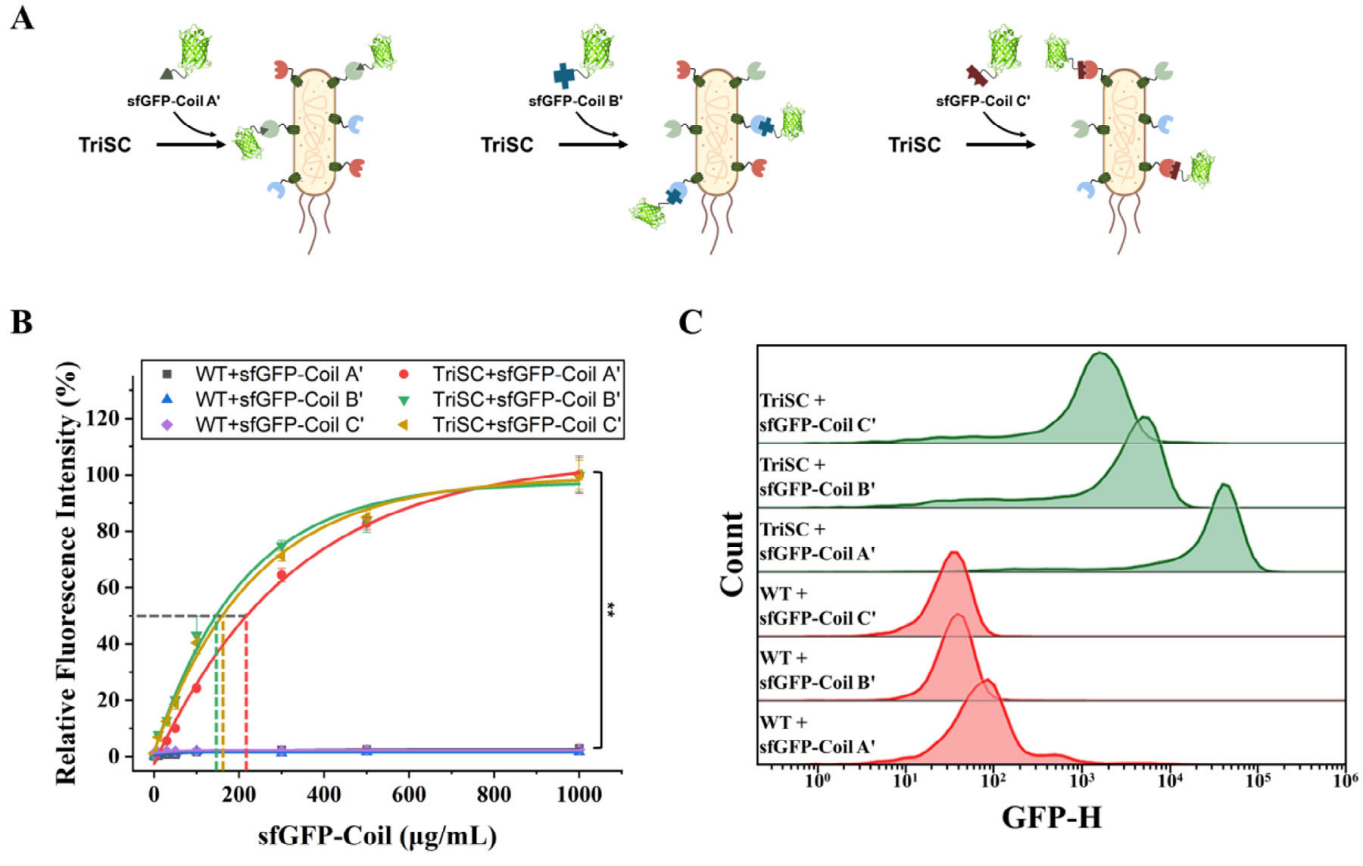
Specific and concentration‐dependent binding of sfGFP–Coil payloads to TriSCs. (A) Schematic representation of selective surface assembly of sfGFP–Coil A′, sfGFP–Coil B′, or sfGFP–Coil C′ on TriSCs through specific coiled‐coil interactions. (B) Relative fluorescence intensities of TriSCs and WT cells after incubation with increasing concentrations of sfGFP–Coil A′, sfGFP–Coil B′, and sfGFP–Coil C′. Fluorescence signals were measured using a microplate reader (Excitation 485 nm / Emission 520 nm). All data shown in B) represent means ± standard deviation (SD) with *n* = 3 (biological replicates). Statistical significance was determined by Welch's t‐test (^*^ *p < *0.05; ^**^ *p < *0.01; ^***^ *p < *0.001; ns, *p* > 0.05). C) Flow cytometry analysis of TriSCs and WT cells treated with sfGFP–Coil A′, sfGFP–Coil B′, or sfGFP–Coil C′.

To demonstrate surface expression of the three coil motifs and their interaction with complementary partners, TriSCs were incubated with three distinct superfolder green fluorescent proteins (sfGFPs) fused to Coil A′, B′, and C′, respectively (Figure 3A). Quantitative total‐cell fluorescence measurements revealed concentration‐dependent increases in signal, with sfGFP–Coil A′ and sfGFP–Coil B′ exhibiting approximately tenfold higher intensities than sfGFP–Coil C′, whereas WT cells showed no detectable fluorescence (Figure S3). When normalized, all sfGFP–Coil payloads exhibited typical ligand–receptor binding curves (Figure 3B). These results indicate that the coil motifs displayed on TriSCs retain their native folds and binding competence toward complementary partners. Moreover, the non‐cooperative independent binding response suggests that TriSCs can be surface‐decorated in a predictable and tunable fashion.

To further assess the surface display of the three coil motifs by single cells, we performed flow cytometry. Forward scatter (FSC) and side scatter (SSC) analyses confirmed that TriSCs maintained cellular characteristics comparable to WT cells (Figure S4A). In the absence of sfGFP–Coil payloads, both TriSCs and WT cells displayed negligible fluorescence (Figure S4B). By contrast, TriSCs treated with sfGFP–Coil A′, sfGFP–Coil B′, or sfGFP–Coil C′ exhibited clear fluorescence shifts, while WT cells showed minimal signals under identical conditions (Figure 3C; Table S4). Notably, sfGFP–Coil C′ produced ∼10‐fold lower mean fluorescence intensity compared to sfGFP–Coil A′ and sfGFP–Coil B′, consistent with weaker C–C′ interactions observed in bulk fluorescence measurements (Figure S3). This difference likely reflects the intrinsically lower affinity of the Coil C–C′ pair relative to A–A′ and B–B′ (Table S3). Collectively, these results validate that all three coil motifs are stably surface‐displayed and remain accessible for specific coiled‐coil recognition, thereby establishing TriSCs as a robust platform for tunable surface functionalization.

### 2.3 Orthogonal and Simultaneous Recruitment of Diverse Payloads

**FIGURE 4:**
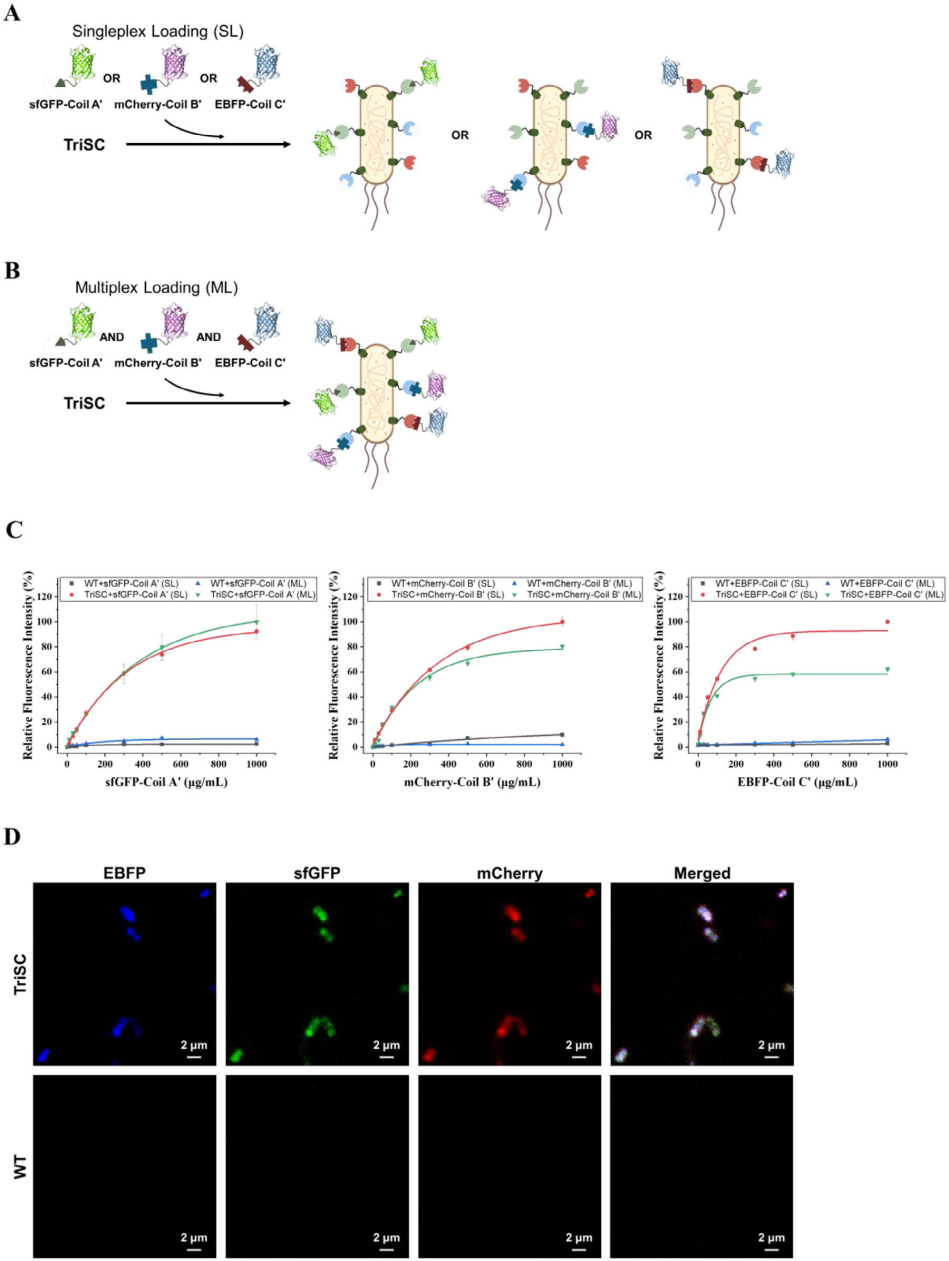
Orthogonal and multiplex assembly of sfGFP–Coil A′, mCherry–Coil B′, and EBFP–Coil C′ with TriSCs. (A) Schematics of singleplex loading (SL) of each fluorescent payload. (B) Schematics of multiplex loading (ML) of all three payloads. (C) Relative mean fluorescence intensities of TriSCs and WT cells under SL and ML conditions. Fluorescence signals were quantified using a microplate reader at fluorophore‐specific excitation/emission settings: 390/460 nm for EBFP, 485/520 nm for sfGFP, and 535/610 nm for mCherry. All data shown in (C) represent means ± standard deviation (SD) from three independent biological replicates (*n* = 3). (D) Confocal microscopy of TriSCs and WT cells treated under ML conditions. For imaging, EBFP, sfGFP, and mCherry were excited at 405, 488, and 555 nm, respectively, with emission detection centered at 435 nm (EBFP), 510 nm (sfGFP), and 580 nm (mCherry). These fluorophore‐specific excitation and emission settings minimize spectral overlap and enable visualization of orthogonal multiplex assembly at the single‐cell level.

To evaluate whether the three coiled‐coil interactions occur independently without cross‐interference, we introduced two additional fluorescent payloads, mCherry–Coil B′ and EBFP–Coil C′, which possess non‐overlapping spectral properties with sfGFP–Coil A′. TriSCs were treated with each payload individually for singleplex loading (Figure 4A, SL) or simultaneously with all three for multiplex loading (Figure 4B, ML) across a range of concentrations. Quantitative total‐cell fluorescence measurements using a microplate reader (390/460 nm for EBFP, 485/520 nm for sfGFP, and 535/610 nm for mCherry) demonstrated that ML binding profiles closely mirrored those observed in SL assays (Figure 4C; Figure S5). While moderate reductions in signal intensity were observed under ML conditions—∼10% for the A–A′ pair, ∼20% for the B–B′ pair, and ∼50% for the C–C′ pair—the overall binding trends were preserved. Because each fluorophore was measured at its optimal excitation and emission wavelengths in the microplate‐based assay, these data provide the primary quantitative assessment of multiplex retention without spectral competition between channels. Confocal microscopy further confirmed the surface‐localized co‐display of all three fluorescent payloads under ML conditions (Figure 4D). In these experiments, EBFP, sfGFP, and mCherry were excited at 405, 488, and 555 nm, respectively, with emission detection centered at 435 nm (EBFP), 510 nm (sfGFP), and 580 nm (mCherry). These fluorophore‐specific excitation and emission settings minimize spectral overlap and cross‐excitation, enabling clear visualization of orthogonal multiplex assembly at the single‐cell level.

To provide an additional quantitative evaluation at the single‐cell level, we performed flow cytometry at a defined protein concentration under compensated conditions (Figure S6 and Table S5). For reliable signal interpretation, fluorescence channels with robust spectral separation under the available laser configuration were selected. EBFP–Coil C′ was analyzed using 405 nm excitation (450/50 nm emission), and sfGFP–Coil A′ was analyzed using 488 nm excitation (530/30 nm emission). Mean fluorescence intensities (MFI) were background‐corrected by subtracting the Cell‐only baseline signal.

Under SL conditions, EBFP–Coil C′ yielded a baseline‐corrected MFI of 1537.96, whereas the corresponding ML value was 698.67, corresponding to ∼45% retention. Similarly, sfGFP–Coil A′ exhibited ∼63% retention under ML relative to SL. These retention values closely match the trends observed in the microplate‐based measurements (Figure 4C), independently confirming that multiplex loading preserves substantial and independent recruitment of each payload, with moderate signal reductions likely arising from steric or surface occupancy effects.

Quantitative flow cytometric analysis of mCherry–Coil B′ was not included due to intrinsic optical constraints of the available instrument configuration. Specifically, mCherry is optimally excited near 561 nm, whereas the flow cytometer relied on 488 nm excitation for red‐channel detection. Under 488 nm excitation, sfGFP exhibits substantially higher effective brightness owing to its higher quantum yield and optimal excitation efficiency, resulting in detectable signal in the red channel that cannot be fully resolved by compensation alone. Indeed, single‐payload controls showed that sfGFP generated stronger apparent red‐channel signal than mCherry under these conditions. To avoid overinterpretation of cross‐excitation artifacts arising from fluorophore brightness differences and excitation mismatch, quantitative comparison was therefore restricted to fluorophores with robust spectral separation.

Collectively, these results demonstrate that the three coiled‐coil pairs function in a highly specific and non‐interfering manner. The consistency between microplate‐based quantification and single‐cell flow cytometry analysis further supports the conclusion that TriSCs enable orthogonal and simultaneous recruitment of multiple payloads with preserved functional independence.

### 2.4 Dynamic and Reversible Assembly via Coiled‐Coil Interactions

**FIGURE 5:**
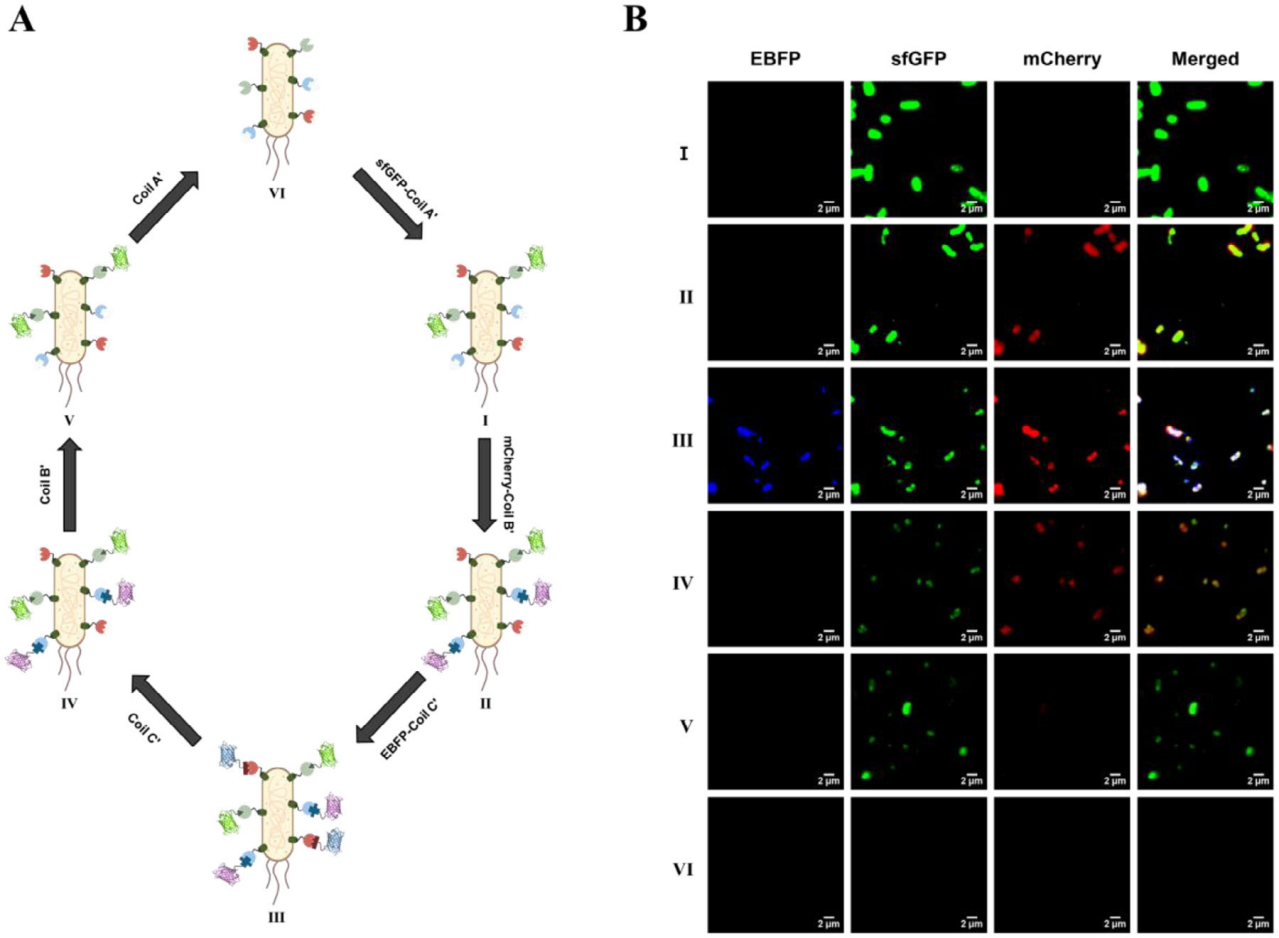
Reversible and orthogonal coiled‐coil assembly of multiple payloads on TriSCs. (A) Schematic of sequential binding and displacement cycles in which fluorescent payloads are recruited to and released from TriSCs. (B) Confocal microscopy images of TriSCs at each step: surface decoration with sfGFP–Coil A′ (I), mCherry–Coil B′ (II), and EBFP–Coil C′ (III); selective displacement of EBFP–Coil C′ (IV), mCherry–Coil B′ (V), and sfGFP–Coil A′ (VI). For imaging, EBFP, sfGFP, and mCherry were excited at 405, 488, and 555 nm, respectively, with emission detection centered at 435 nm (EBFP), 510 nm (sfGFP), and 580 nm (mCherry).

To examine whether payload binding is reversible and switchable in a temporally controlled manner, we performed sequential binding and displacement assays (Figure 5A). TriSCs were first decorated stepwise with sfGFP–Coil A′, mCherry–Coil B′, and EBFP–Coil C′, yielding distinct green, red, and blue surface fluorescence (Figures 5B, I–III). Subsequent addition of competitor peptides selectively displaced each corresponding fluorophore in sequence, ultimately restoring cells to a non‐fluorescent state (Figures 5B, IV–VI). These results demonstrate that coiled‐coil interactions on TriSCs are reversible, orthogonal, and temporally controllable, enabling programmable assembly and removal of multiple payloads.

### 2.5 Programmable Live Scaffolding for Spatially Coordinated Targeting and Synergistic Apoptosis

We next investigated whether TriSCs could function as programmable live‐cell scaffolds that enable conditional synergy between targeting and therapeutic nanobodies. Specifically, we tested the hypothesis that synergistic cytotoxic activity between DR5 Nb3–Coil C′ and αEGFR Nb–Coil B′ emerges only when the two nanobodies are spatially colocalized on a common scaffold (TriSC), rather than when they are simply co‐administered in solution.

Death receptor 5 (DR5) activation induces apoptosis via assembly of the death‐inducing signaling complex (DISC), representing a well‐established anticancer mechanism [54, 55]. In contrast, epidermal growth factor receptor (EGFR), frequently overexpressed in colorectal cancer cells, serves as a surface anchor capable of stabilizing carrier–cell interactions [56, 57, 58]. Accordingly, an agonistic DR5 nanobody (DR5 Nb3) and an EGFR‐binding nanobody (αEGFR Nb) were genetically fused to Coil C′ and Coil B′, respectively, enabling orthogonal and modular surface display on TriSCs.

**FIGURE 6:**
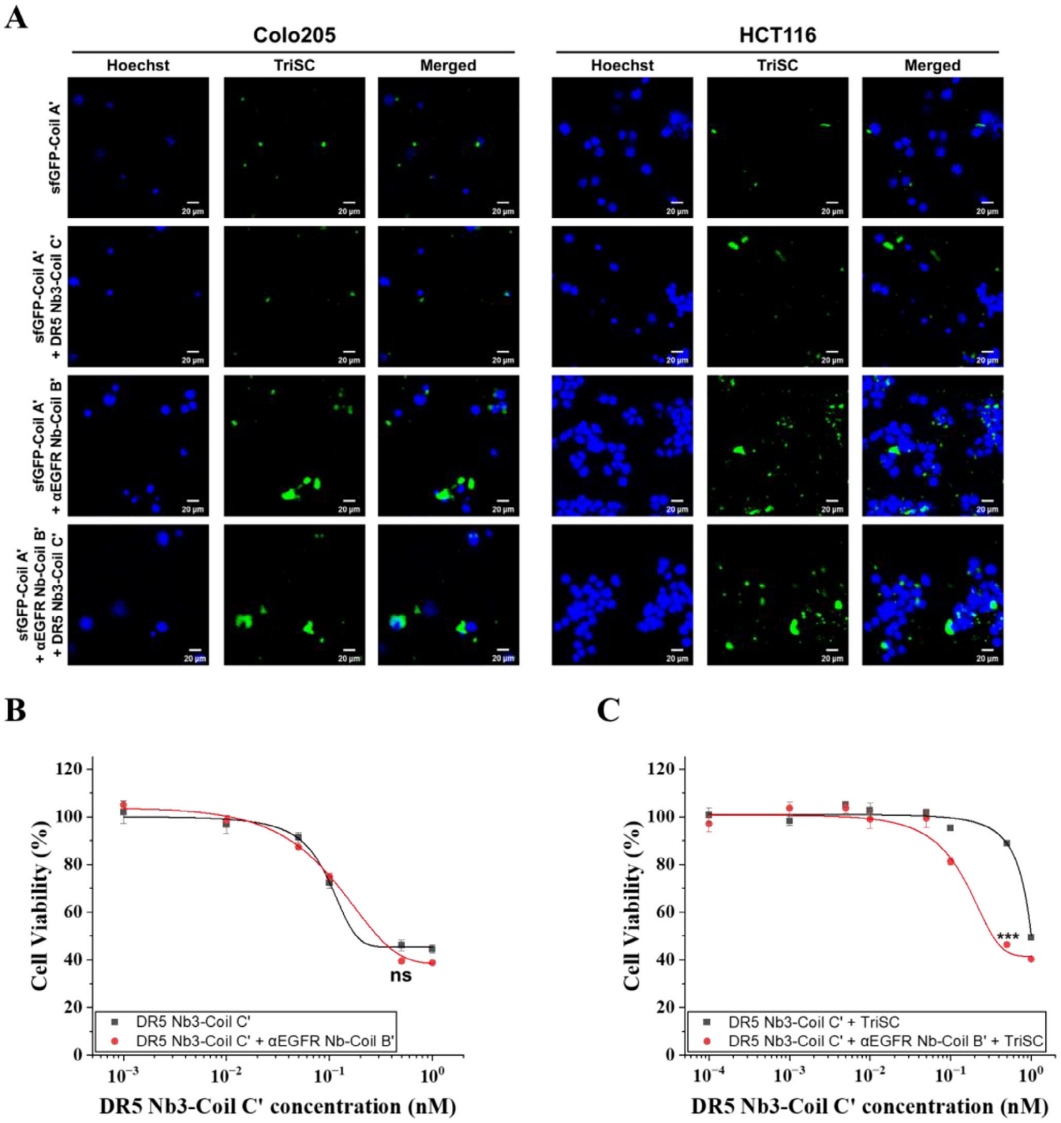
TriSC–mediated colocalization of targeting and therapeutic nanobodies. (A) Confocal microscopy analysis of Colo205 and HCT116 colorectal cancer cells incubated with TriSCs (1.0 × 10^5^ cells per well) displaying sfGFP–Coil A′ alone, DR5 Nb3–Coil C′, αEGFR Nb–Coil B′, or their combinations. Cell nuclei were stained with Hoechst (blue), TriSCs were visualized via sfGFP fluorescence (green), and merged images are shown. (B) Viability of Colo205 cells after treatment with serial concentrations of DR5 Nb3–Coil C′ in the absence or presence of soluble αEGFR Nb–Coil B′ (100 nm). Cell viability was measured after 24 h using a WST‐8 assay. (C) Viability of Colo205 cells after treatment with TriSCs (10^5^ cells) displaying DR5 Nb3–Coil C′ in the absence or presence of αEGFR Nb–Coil B′ (100 nm). Data are shown as mean ± SD (*n* = 3 biological replicates). Statistical significance was determined by Welch's t‐test (^*^ *p* < 0.05; ^**^ *p* < 0.01; ^***^ *p* < 0.001; ns, not significant).

We first examined the role of αEGFR Nb–Coil B′ in cancer cell targeting using TriSCs displaying sfGFP–Coil A′ as a fluorescent reporter. Confocal microscopy revealed that TriSCs displaying sfGFP–Coil A′ alone exhibited only weak association with Colo205 and HCT116 colorectal cancer cells, both of which express moderate levels of EGFR and DR5 [59, 60, 61, 62, 63], likely reflecting the intrinsic tumor‐tropic properties of the *E. coli* Nissle 1917 chassis (Figure 6A). Display of DR5 Nb3–Coil C′ resulted in minimal enhancement of binding, consistent with the relatively modest affinity of DR5 engagement [64]. In contrast, TriSCs displaying αEGFR Nb–Coil B′ showed robust and sustained surface association with EGFR‐positive cancer cells, which was preserved upon co‐display of DR5 Nb3–Coil C′. These results establish αEGFR Nb–Coil B′ as the principal determinant of cancer cell targeting, while DR5 Nb3–Coil C′ functions primarily as the cytotoxic effector module.

To determine whether simple co‐administration of targeting and therapeutic nanobodies is sufficient to induce synergistic cytotoxicity, Colo205 cells were treated with serial concentrations of DR5 Nb3–Coil C′ in the presence or absence of soluble αEGFR Nb–Coil B′ (100 nM). αEGFR Nb‐Coil B′ alone did not induce significant cytotoxicity, affirming its tolerability (Figure S7). While DR5 Nb3–Coil C′ alone reduced cell viability in a dose‐dependent manner, addition of soluble αEGFR Nb–Coil B′ did not further enhance cytotoxicity, yielding a dose–response curve indistinguishable from that of DR5 Nb3–Coil C′ alone (Figure 6B). This result indicates that, in the absence of spatial coordination, targeting and therapeutic nanobodies do not cooperate to enhance DR5‐mediated killing.

We next evaluated whether colocalization of these nanobodies on TriSCs could alter functional outcomes. TriSCs were incubated with DR5 Nb3–Coil C′ at varying concentrations in the presence or absence of αEGFR Nb–Coil B′ (100 nm) and directly applied to Colo205 cells under dose‐matched conditions. TriSCs alone elicited no discernible cytotoxicity, demonstrating their biocompatibility (Figure S7). In contrast to soluble nanobody mixtures, co‐display of DR5 Nb3–Coil C′ and αEGFR Nb–Coil B′ on TriSCs resulted in a pronounced enhancement of cytotoxicity, manifested as a leftward shift of the dose–response curve relative to TriSCs displaying DR5 Nb3–Coil C′ alone (Figure 6C). Although the absolute IC50 values observed in TriSC–based conditions were higher than those obtained with freely diffusing nanobodies, this modest rightward shift likely reflects the spatially confined and interface‐restricted mode of action imposed by surface immobilization, rather than diminished intrinsic DR5 agonistic potency.

We propose that αEGFR Nb–mediated engagement of EGFR stabilizes TriSC–cancer cell interactions, thereby creating a confined cell–cell interface. Within this interface, surface‐immobilized DR5 Nb3 can cluster DR5 receptors at high local density, promoting efficient DISC assembly and apoptotic signaling. Such spatially coordinated receptor engagement is not achievable with freely diffusing nanobodies and explains why synergistic killing emerges exclusively in the TriSC–based format. Importantly, this synergy is achieved without genetic fusion of targeting and effector domains into a single bispecific molecule. Instead, independent nanobody modules are assembled onto TriSCs in a mix‐and‐go manner through orthogonal and spontaneous coiled‐coil interactions, enabling rapid and modular integration of targeting and effector functions. This configuration functionally parallels multispecific biologics while mitigating interdomain folding constraints and the structural and manufacturing complexity inherent to genetically fused bispecific constructs [65, 66, 67].

Collectively, these findings demonstrate that TriSCs function as programmable live‐cell scaffolds that convert spatially localized targeting into amplified apoptotic signaling through nanobody colocalization. Beyond this specific DR5/EGFR example, the platform enables facile implementation of combinatorial, spatially coordinated multi‐drug strategies, rather than simple cocktail administration, underscoring its potential as a versatile format for modular and synergistic therapeutic design.

## 3 Conclusion

In this study, we developed TriSCs, a tri‐modular live biotherapeutic platform that enables programmable, reversible, and tunable multiplex surface assembly through orthogonal coiled‐coil interactions. Systematic characterization confirmed that three distinct motifs were robustly displayed, retained binding specificity, and operated without cross‐interference. Sequential displacement assays further demonstrated that payloads could be dynamically added or removed in a temporally controlled and reversible manner, distinguishing this system from covalent or genetically encoded strategies.

Building on this versatility, we equipped TriSCs with therapeutic and targeting nanobodies. Co‐display of a DR5 agonist nanobody and an EGFR‐binding nanobody yielded synergistic anticancer activity not achievable with soluble mixtures, demonstrating that the TriSC platform enables cooperative therapeutic effects. In parallel, incorporation of a fluorescent reporter enabled real‐time bioimaging, illustrating the multifunctionality of the system.

While the present study focuses on establishing a mechanistic and proof‐of‐concept framework under controlled in vitro conditions, these results provide a solid foundation for future in vivo evaluation. In particular, the modularity, reversibility, and tunability of TriSCs offer unique opportunities to optimize payload composition, dosing sequence, and functional balance prior to animal studies. Such in vivo investigations will be an important next step toward assessing biodistribution, safety, and therapeutic efficacy in physiologically relevant settings.

Together, these results present TriSCs as a programmable and reconfigurable scaffold integrating targeting, therapy, and imaging, thereby advancing next‐generation bioengineered LBPs.

## 4 Experimental Section

### 4.1 Materials

*E. coli* Nissle 1917 was kindly provided as a gift from Gyoo Yeol Jung's lab in POSTECH. *E. coli* SHuffle T7 (#C3026J) was purchased from New England Biolabs (Ipswich, MA). The gene for the anti‐DR5 nanobody (11H6) was obtained from patent [68], and the gene for the anti‐EGFR nanobody (7D12) was described by Roovers et al. [69]. Ni‐NTA agarose was provided from Qiagen (Valencia, CA). Amicon Ultra centrifugal filters with a cutoff of 10 kDa and Millicell EZ SLIDE 8‐well glass chambers were bought from Merck Corporation (Darmstadt, Germany). PD‐10 desalting columns were obtained from GE Healthcare (Piscataway, NJ). The Mini‐PROTEAN system for polyacrylamide electrophoresis was obtained from Bio‐Rad (Hercules, CA). A hand‐cast gel was made according to a standard protocol for a 12% polyacrylamide gel. Plasmid extraction and gel extraction kits were purchased from Bioneer (Daejeon, Korea). Sodium chloride, sodium phosphate monobasic and sodium phosphate dibasic were purchased from Samchun Chemical (Seoul, Korea). Purified Mouse anti‐6×His primary antibody (#MA1‐21315), purified Mouse anti‐FLAG tag antibody (#MA1‐91878), purified Mouse anti‐HA tag antibody (#26183) and Hoechst 33342 were purchased from Thermo Fisher Scientific (Waltham, MA). Goat anti‐Mouse IgG (H+L)‐HRP was purchased from GenDEPOT (Altair, TX). Untagged Coil A′, Coil B′, and Coil C′ peptides were synthesized from GL Biochem (Shanghai, China). RPMI‐1640, fetal bovine serum, and penicillin‐streptomycin were purchased from Thermo Fisher Scientific (Waltham, MA). All other chemicals and reagents were purchased from Sigma‐Aldrich (St. Louis, MO).

### 4.2 Cell Culture

HCT116 (#10247), and Colo205 (#10222) cell lines were obtained from the Korean Cell Line Bank (Seoul, Korea). Cells were maintained in RPMI‐1640 medium supplemented with 10% fetal bovine serum and 1% penicillin‐streptomycin and cultured at 37°C in a humidified incubator with 5% CO_2_.

### 4.3 Gene Cloning and Plasmid Construction

Genes encoding Lpp, OmpA, sfGFP‐(G_4_S)_3_ with an N‐terminal hexahistidine tag, mCherry, EBFP, DR5 Nb3, αEGFR Nb, and selected coils were synthesized by Bionics (Korea). All cloning procedures were carried out using the In‐Fusion HD Cloning Kit (Takara, Japan), which enables seamless assembly of PCR‐amplified inserts with linearized vectors through 18‐bp homologous recombination. Specifically, to achieve comparable expression levels with Lpp‐OmpA‐Coil B and Lpp‐OmpA‐Coil C expressed from the pQE80‐T5 vector, an expression vector pRSF1‐T5 was constructed by replacing the original T7 promoter and T7 terminator in pRSF1b (#71363‐3, Merck) with the T5 promoter and lambda t0 terminator. Then, to construct expression plasmids pRSF1‐T5‐Lpp‐OmpA‐Coil A‐His, Lpp‐OmpA and Coil A were PCR‐amplified with appropriate 18 bp overhangs and assembled into linearized pRSF1‐T5 vectors using In‐Fusion cloning. For the construction of pQE80‐T5‐Lpp‐OmpA‐Coil B‐FLAG and Lpp‐OmpA‐Coil C‐HA, Lpp‐OmpA and Coil B were first inserted into pQE80L (#VET1018, Creative Biogene) as described above. Subsequently, Lpp‐OmpA and coil C were PCR‐amplified and introduced into a linearized pQE80‐Lpp‐OmpA‐Coil B backbone, generating pQE80 coil expression vectors. As a control, pQE80 w/o coil expression vectors were also constructed by inserting only the Lpp‐OmpA fragment into linearized pQE80 vectors. To generate the payload constructs, sfGFP‐(G_4_S)_3_ and complementary coil motifs A′, B′, and C′ were amplified and inserted into linearized pQE80, resulting in pQE80‐sfGFP‐Coil A′, pQE80‐Coil B′, and pQE80‐Coil C′, respectively. The sfGFP gene was then replaced as appropriate with mCherry, EBFP, DR5 Nb3, or αEGFR Nb to create a library of coil tagged fluorescent and nanobody payloads. Every construct was confirmed by Sanger sequencing. Information about amino acid sequences and primers used in this study are provided in Tables S1 and S2, respectively.

### 4.4 Expression and Purification of TriSCs and Payloads

Both pRSF1 coil expression vectors and pQE80 coil expression vectors were co‐transformed into EcN, resulting in TriSCs. In contrast, pQE80 w/o coil expression vectors were transformed into EcN, to produce WT control cells. Both TriSCs and WT cells were initially cultured to saturation at 37°C in 2×YT medium supplemented with 100 µg mL^−1^ ampicillin and 50 µg mL^−1^ kanamycin under vigorous shaking. The saturated cultures were then diluted 100 times into fresh 2×YT medium to initiate the main culture. When the optical density at 600 nm (OD600) reached approximately 0.6, protein expression was induced by 1 mM IPTG and the temperature was adjusted to 30°C. After 4 h of induction, both TriSCs and WT cells were harvested, washed 3 times with PBS, and resuspended in PBS. Similarly, *E. coli* SHuffle strains containing the payload expression vectors were cultivated at 18°C following induction with 1 mm IPTG. After 30 h of cultivation, cells were harvested by centrifugation and resuspended in lysis buffer containing 50 mm phosphate buffer (pH 8.0), 300 mm NaCl, 10 mm imidazole, and 1 mg mL^−1^ lysozyme. The cell suspension was incubated at 4°C with gentle rotation for 3 min to facilitate cell wall degradation. Subsequent lysis was performed by sonication (10 s on/10 s off) for 10 min, followed by centrifugation at 12 000 rpm for 30 min at 4°C to remove cell debris. The resulting supernatant was filtered through a 0.45 µm membrane and incubated with Ni‐NTA agarose resin for 30 min at 4°C to allow His‐tagged protein binding. The resin was then washed on a gravity‐flow column using phosphate buffer (pH 8.0) containing 30 mm imidazole to remove non‐specifically bound proteins. Bound proteins were eluted using phosphate buffer (pH 8.0) supplemented with 250 mm imidazole. Eluted fractions were buffer‐exchanged to PBS (pH 7.4) using PD‐10 desalting columns, aliquoted, and stored at −20°C until use. SDS–PAGE and FPLC analyses confirmed high purity of the proteins (Figure S8).

### 4.5 Western Blot

Proteins were resolved by 12% SDS‐PAGE under reducing conditions and transferred onto polyvinylidene difluoride (PVDF) membranes using the iBlot 2 Gel Transfer Device (Thermo Fisher Scientific, US). Membranes were blocked for 2 h at room temperature (RT) in PBST (PBS with 0.5% v/v Tween 20) containing 5% w/v skim milk. Subsequently, membranes were incubated for 2 h at RT with one of the primary antibodies (1:5000). The primary antibodies used were anti‐6×His (Thermo Fisher Scientific, #MA1‐21315), anti‐FLAG (Thermo Fisher Scientific, #MA1‐91878), or anti‐HA (Thermo Fisher Scientific, #26183). Following primary antibody incubation, the membranes were washed three times for 5 min with PBST. Membranes were then incubated with appropriate HRP‐conjugated secondary antibodies (1:10000, GenDEPOT, #SA001‐500) for 1.5 h at RT. Final washes were performed three times for 10 min. Signal detection was conducted using ECL reagent and visualized with Davinch‐Chemi Fluoro Imaging system (Davinch‐K, Korea).

### 4.6 Fluorescence Measurements

A total of 1 × 10^6^ TriSCs or WT cells were incubated with sfGFP–Coil A′, sfGFP–Coil B′ or sfGFP–Coil C′ at concentrations of 0, 10, 30, 50, 100, 300, 500, 1000 µg mL^−1^ for 30 min at RT. Following incubation, the cells were washed twice with PBS by centrifugation to remove unbound proteins, and fluorescence was measured at an excitation wavelength of 485 nm and an emission wavelength of 520 nm. In a similar manner, a total of 1 × 10^6^ TriSCs or WT cells were incubated with sfGFP–Coil A′, mCherry–Coil B′, or EBFP–Coil C′ either individually or in combination, using the same concentration range and conditions as described above. After incubation and washing, each fluorescence intensity was measured. Specifically, EBFP was analyzed at an excitation wavelength of 390 nm and an emission of 460 nm, GFP at an excitation of 485 nm and an emission of 520 nm, mCherry at an excitation of 535 nm and an emission of 610 nm. Each experiment was carried out in triplicate. Graphs were fitted using Asymptotic 1 model in Origin 2025 to calculate K_D_ values.

### 4.7 Flow Cytometry

To evaluate surface functionalization of TriSCs, a total of 1.0 × 10^6^ TriSCs or WT cells were incubated without payloads or with sfGFP–Coil A′, sfGFP–Coil B′, or sfGFP–Coil C′ at a final concentration of 0.5 mg mL^−1^ in 100 µL PBS for 30 min at RT. Each sample was washed twice with PBS and resuspended in 1 mL PBS. Flow cytometry analysis was performed using BD FACSVerse Cell Analyzer (BD Biosciences, US) equipped with BD FACSuite software. Forward scatter (FSC) and side scatter (SSC) signals were acquired using a 488 nm laser and a 488/10 nm bandpass filter. GFP fluorescence was excited by a blue laser (488 nm) and detected using a 510/10 nm bandpass filter. The FSC and SSC detectors were set to 727.5 V and 521.0 V, respectively, and the GFP detector was set to 450 V. For each sample, 10 000 ungated events were recorded prior to gating, and bacterial populations were distinguished from background debris based on FSC and SSC profiles.

To quantitatively evaluate singleplex (SL) and multiplex (ML) payload recruitment at the single‐cell level, TriSCs or WT cells (1.0 × 10^6^) were incubated with fluorescent payload proteins at 0.5 mg mL^−1^ under SL or ML conditions, followed by washing with PBS to remove unbound proteins prior to analysis. Data acquisition was performed using a flow cytometer equipped with 405 nm and 488 nm excitation lasers. EBFP–Coil C′ was analyzed using 405 nm excitation with a 450/50 nm emission filter. sfGFP–Coil A′ was analyzed using 488 nm excitation with a 530/30 nm emission filter. For red‐channel detection (mCherry–Coil B′), 488 nm excitation with a 700/54 nm emission filter was used, consistent with the available instrument configuration.

For each sample, 10 000 events were collected. Bacterial populations were gated based on FSC and SSC to exclude debris. SL controls (cells incubated with individual payloads) were used to verify channel specificity and to assess spectral spillover. Compensation was applied according to the standard matrix‐based compensation procedure of the instrument.

Mean fluorescence intensity (MFI) values were extracted for each gated population. Background correction was performed by subtracting the corresponding TriSC–only control MFI from each sample:

$$\;MFI_{corrected}=MFI_{sample}-\;MFI_{cell-only}$$

For comparison between SL and ML conditions, retention (%) was calculated as:

$$Retention\;\left(\%\right)=\frac{MFI_{ML,corrected}\;}{MFI_{SL,corrected}}\;\times 100$$

WT cells treated with identical payload conditions were included as negative controls to assess nonspecific binding and autofluorescence.

Due to the optical configuration of the instrument, quantitative analysis was restricted to fluorophores with robust spectral separation. Specifically, EBFP–Coil C′ and sfGFP–Coil A′ were quantitatively analyzed. mCherry–Coil B′ was not subjected to quantitative retention analysis because mCherry is optimally excited at approximately 561 nm, whereas the available instrument relied on 488 nm excitation for red‐channel detection. Under 488 nm excitation, sfGFP exhibits substantially higher effective brightness owing to its higher quantum yield and optimal excitation efficiency, resulting in detectable emission extending into the red detection channel. SL controls confirmed that sfGFP generated stronger apparent signal in the red channel than mCherry under these conditions. Because such excitation mismatch and differences in fluorophore brightness imbalance cannot be fully resolved by compensation alone, quantitative interpretation of mCherry‐derived signals was not performed to avoid overestimation due to cross‐excitation artifacts. All flow cytometry data are presented as MFI ± standard deviation (SD).

### 4.8 Confocal Microscopy

To visualize multiplexed surface assembly, a total of 1.0 × 10^7^ TriSCs or WT cells were incubated with a mixture of sfGFP–Coil A′, mCherry–Coil B′, and EBFP–Coil C′ at a concentration of 0.5 mg mL^−1^ for each payload in 100 µL PBS for 30 min at RT. Then samples were washed twice with PBS and resuspended in 100 µL PBS. For microscopy, 5 µL of each cell suspension was placed on a glass slide, sealed with a coverslip, and left undisturbed for 30 min to allow cells to settle. Fluorescence imaging was performed using a ZEISS LSM700 confocal laser scanning microscope (Carl Zeiss, Germany) under a 63× oil immersion objective. EBFP fluorescence was excited using a 405 nm diode laser (5 mW) operated at 53.2 arbitrary units of laser power, and emission was collected at 435 nm using a PMT gain of 1052. sfGFP fluorescence was excited with a 488 nm diode laser (10 mW) at a laser power setting of 5.2, and emission was detected at 510 nm with a PMT gain of 865. mCherry fluorescence was excited using a 555 nm diode laser (10 mW) at a laser power of 11.9, and emission was collected at 580 nm with a PMT gain of 842.

To evaluate targeting efficiency, HCT116 cells or Colo205 cells were seeded at a density of 2.0 × 10^5^ cells mL^−1^ in the Millicell EZ SLIDE 8‐well glass chambers and incubated at 37°C for 24 h. Then, 5.0 × 10^5^ TriSCs were pre‐incubated with 10 nM of sfGFP–Coil A′ in the presence or absence of 100 nM of αEGFR‐Coil B′ and 10 nm of DR5 Nb3–Coil C′ for 30 min. The labeled TriSCs were then co‐incubated with cultured target cells for 6 h. After incubation, cancer cells were stained with 5 µg mL^−1^ of Hoechst 33342 for 15 min, washed three times with PBS, and analyzed by confocal microscopy. Hoechst 33342 was excited at 405 nm diode laser with a laser power setting of 65.0, and emission was detected at 450 nm with a PMT gain of 969.

### 4.9 Orthogonal and Reversible Binding Assay

To evaluate the reversibility and modularity of coiled‐coil surface functionalization, TriSCs were sequentially incubated with fluorescently labeled payloads and competitor peptides under controlled conditions. All reactions were carried out in 100 µL PBS at RT, and each intermediate state was prepared as an individual sample for a confocal microscope. First, 1.0 × 10^7^ TriSCs were incubated with 30 µg of sfGFP–Coil A′ for 30 min, followed by a PBS wash and centrifugation at 13 000 rpm for 5 min. The cell pellet was resuspended in PBS and collected as the first sample to confirm GFP binding. Samples were then incubated with 100 µg of mCherry‐Coil B′ under identical conditions. After washing and resuspension, the second sample was collected to evaluate dual fluorescence from GFP and mCherry. Next, 100 µg of EBFP–Coil C′ was added to the suspension, and after incubation, washing, and resuspension, the third sample was obtained to confirm successful triple payload binding. To investigate selective dissociation, cells were subsequently treated with 100 µg of the non‐tagged Coil C′ peptide for 1 h to competitively displace EBFP–Coil C′. After washing and resuspension, the fourth sample was collected to evaluate the loss of EBFP signal while retaining GFP and mCherry fluorescence. The same process was repeated using 100 µg of non‐tagged Coil B′ peptide to displace mCherry–Coil B′, and the fifth sample was collected to detect remaining GFP fluorescence. Finally, 100 µg of non‐tagged Coil A′ peptide was added to displace sfGFP‐Coil A′. After incubation and washing, the final sample was collected to confirm the complete removal of all bound payloads. All samples were mounted on glass slides and imaged using ZEISS LSM700 confocal microscopy with previously described settings to monitor stepwise assembly and disassembly of fluorescent signals on the TriSC surface.

### 4.10 In Vitro Cell Viability

Colo205 cells obtained from the Korean Cell Line Bank (KCLB No: 10222) were seeded in 96‐well microplates at a density of 1.0 × 10^4^ cells per well and incubated for 24 h. Serial concentrations of DR5 Nb3–Coil C′ were preincubated for 30 min in the presence or absence of 100 nM αEGFR Nb–Coil B′ and/or 1.0 × 10^5^ TriSCs. The resulting mixtures were directly applied to the cultured cells without an intermediate washing or isolation step and incubated for an additional 24 h. This design was adopted to maintain consistent bulk nanobody exposure across experimental conditions. Cell viability was assessed using 10% WST‐8 reagent (GenDEPOT, US). After incubation at 37°C for 1–4 h, absorbance at 450 nm was measured using a Hidex Sense multimodal microplate reader (Hidex, Finland). Dose–response curves were fitted using a nonlinear dose–response model in Origin 2025.

### 4.11 Statistical Analysis

All data are presented as mean ± SD. Unless otherwise indicated, experiments were performed in triplicate (*n* = 3 independent biological replicates). For flow cytometry analysis, 10 000 events were collected per sample. For comparisons among multiple groups, one‐way analysis of variance (ANOVA) was used where appropriate. For pairwise comparisons between two groups, Welch's two‐tailed unpaired t‐test was applied to account for potential unequal variances. A two‐sided testing approach was used throughout the study. A *p* value < 0.05 was considered statistically significant (^*^ *p* < 0.05; ^**^ *p* < 0.01; ^***^ *p* < 0.001; ns, *p* ≥ 0.05). Dissociation constants (K_D_) were determined by nonlinear regression using the Asymptotic 1 model in Origin 2025. Dose–response curves were fitted using nonlinear regression in Origin 2025.

## Conflicts of Interest

The authors declare no conflicts of interest.

## Supporting information




**Supporting File**: smll73196‐sup‐0001‐SuppMat.docx.

## Data Availability

The data that support the findings of this study are available from the corresponding author upon reasonable request.
