# Supplementary material for: Orthogonal Tri‐Modular Coiled‐Coil Assembly for Programmable Multi‐Cargo Display on Escherichia coli Nissle 1917
Source: Small. 2026 Mar 20;22(28):e12642. doi: 10.1002/smll.202512642 (PMC13181507; doi:10.1002/smll.202512642)
Supplement: Supplementary file 1 — Supporting File: smll73196‐sup‐0001‐SuppMat.docx. [file SMLL-22-e12642-s001.docx]

**Table S1.** Information of amino acid sequences.

| **Name** | **Sequence** | **Length**  **(AA)** |
| --- | --- | --- |
| Lpp signal peptide-Lpp-OmpA-  Coil A-His Tag | MKATKLVLGAVILGSTLLAGCSSNAKIDQGINPYVGFEMGYDWLGRMPYKGQRENGAYKAQGVQLTAKLGYPITDDLDIYTRLGGMVWRADTKSNVYGKNHDTGVSPVFAGGVEYAITPEIATRLEYQWTNNIGDAHTIGTRPDNGGGSGGGSLEIEAAALEQENTALETEVAELEQEVQRLENIVSQYRTRYGPLHHHHHH | 202 |
| Lpp signal peptide-Lpp-OmpA-  Coil B-FLAG Tag | MKATKLVLGAVILGSTLLAGCSSNAKIDQGINPYVGFEMGYDWLGRMPYKGQRENGAYKAQGVQLTAKLGYPITDDLDIYTRLGGMVWRADTKSNVYGKNHDTGVSPVFAGGVEYAITPEIATRLEYQWTNNIGDAHTIGTRPDNGGGSGGGSEVSALEKEVSALEKEVSALEKEVSALEKEVSALEKDYKDDDDK | 196 |
| Lpp signal peptide-Lpp-OmpA-  Coil C-HA Tag | MKATKLVLGAVILGSTLLAGCSSNAKIDQGINPYVGFEMGYDWLGRMPYKGQRENGAYKAQGVQLTAKLGYPITDDLDIYTRLGGMVWRADTKSNVYGKNHDTGVSPVFAGGVEYAITPEIATRLEYQWTNNIGDAHTIGTRPDNGGGSGGGSKAFIVTDEDIRKQEERVQQVRKKLEEALMADILSYPYDVPDYA | 196 |
| Lpp signal peptide-Lpp-OmpA | MKATKLVLGAVILGSTLLAGCSSNAKIDQGINPYVGFEMGYDWLGRMPYKGQRENGAYKAQGVQLTAKLGYPITDDLDIYTRLGGMVWRADTKSNVYGKNHDTGVSPVFAGGVEYAITPEIATRLEYQWTNNIGDAHTIGTRPDN | 145 |
| His Tag-sfGFP-  Coil A' | MHHHHHHSKGEELFTGVVPILVELDGDVNGHKFSVRGEGEGDATNGKLTLKFICTTGKLPVPWPTLVTTLTYGVQCFSRYPDHMKRHDFFKSAMPEGYVQERTISFKDDGTYKTRAEVKFEGDTLVNRIELKGIDFKEDGNILGHKLEYNFNSHNVYITADKQKNGIKANFKIRHNVEDGSVQLADHYQQNTPIGDGPVLLPDNHYLSTQSVLSKDPNEKRDHMVLLEFVTAAGITHGMDELYKGGGGSGGGGSGGGGSLEIRAAALRRRNTALRTRVAELRQRVQRLRNEVSQYETRYGPL | 302 |
| His Tag-sfGFP-  Coil B' | MHHHHHHSKGEELFTGVVPILVELDGDVNGHKFSVRGEGEGDATNGKLTLKFICTTGKLPVPWPTLVTTLTYGVQCFSRYPDHMKRHDFFKSAMPEGYVQERTISFKDDGTYKTRAEVKFEGDTLVNRIELKGIDFKEDGNILGHKLEYNFNSHNVYITADKQKNGIKANFKIRHNVEDGSVQLADHYQQNTPIGDGPVLLPDNHYLSTQSVLSKDPNEKRDHMVLLEFVTAAGITHGMDELYKGGGGSGGGGSGGGGSKVSALKEKVSALKEKVSALKEKVSALKEKVSALKE | 294 |
| His Tag-sfGFP-  Coil C' | MHHHHHHSKGEELFTGVVPILVELDGDVNGHKFSVRGEGEGDATNGKLTLKFICTTGKLPVPWPTLVTTLTYGVQCFSRYPDHMKRHDFFKSAMPEGYVQERTISFKDDGTYKTRAEVKFEGDTLVNRIELKGIDFKEDGNILGHKLEYNFNSHNVYITADKQKNGIKANFKIRHNVEDGSVQLADHYQQNTPIGDGPVLLPDNHYLSTQSVLSKDPNEKRDHMVLLEFVTAAGITHGMDELYKGGGGSGGGGSGGGGSPEERERMIKQLKEELRLEEAKLVLLKKLRQSQIQKEATAQK | 300 |
| His Tag-mCherry-  Coil B' | MHHHHHHVSKGEEDNMAIIKEFMRFKVHMEGSVNGHEFEIEGEGEGRPYEGTQTAKLKVTKGGPLPFAWDILSPQFMYGSKAYVKHPADIPDYLKLSFPEGFKWERVMNFEDGGVVTVTQDSSLQDGEFIYKVKLRGTNFPSDGPVMQKKTMGWEASSERMYPEDGALKGEIKQRLKLKDGGHYDAEVKTTYKAKKPVQLPGAYNVNIKLDITSHNEDYTIVEQYERAEGRHSTGGMDELYKGGGGSGGGGSGGGGSKVSALKEKVSALKEKVSALKEKVSALKEKVSALKE | 292 |
| His Tag-EBFP-  Coil C' | MHHHHHHVSKGEELFTGVVPILVELDGDVNGHKFSVRGEGEGDATNGKLTLKFICTTGKLPVPWPTLVTTLSHGVQCFARYPDHMKQHDFFKSAMPEGYVQERTIFFKDDGTYKTRAEVKFEGDTLVNRIELKGVDFKEDGNILGHKLEYNFNSHNIYIMAVKQKNGIKVNFKIRHNVEDGSVQLADHYQQNTPIGDGPVLLPDSHYLSTQSVLSKDPNEKRDHMVLLEFRTAAGITLGMDELYKGGGGSGGGGSGGGGSPEERERMIKQLKEELRLEEAKLVLLKKLRQSQIQKEATAQK | 301 |
| His Tag-αEGFR Nb-  Coil B' | MHHHHHHQVKLEESGGGSVQTGGSLRLTCAASGRTSRSYGMGWFRQAPGKEREFVSGISWRGDSTGYADSVKGRFTISRDNAKNTVDLQMNSLKPEDTAIYYCAAAAGSAWYGTLYEYDYWGQGTQVTVSSGGGGSGGGGSGGGGSKVSALKEKVSALKEKVSALKEKVSALKEKVSALKE | 181 |
| His Tag-DR5 Nb3-  Coil C' | MHHHHHHEVQLVESGGGLVQPGGSLRLSCAASGTFDKINNMGWYRQAPGKQRDLVAQITPGGITDYADSVKGRFTISRDNAKDTMYLQMNSLKPEDTAVYFCNAEILKRAYIDVYVNYWGQGTQVTVSSGGGGSGGGGSGGGGSEVQLVESGGGLVQPGGSLRLSCAASGTFDKINNMGWYRQAPGKQRDLVAQITPGGITDYADSVKGRFTISRDNAKDTMYLQMNSLKPEDTAVYFCNAEILKRAYIDVYVNYWGQGTQVTVSSGGGGSGGGGSGGGGSGGGGSEVQLVESGGGLVQPGGSLRLSCAASGTFDKINNMGWYRQAPGKQRDLVAQITPGGITDYADSVKGRFTISRDNAKDTMYLQMNSLKPEDTAVYFCNAEILKRAYIDVYVNYWGQGTQVTVSSGGGGSGGGGSGGGGSPEERERMIKQLKEELRLEEAKLVLLKKLRQSQIQKEATAQK | 464 |

**Table S2.** Primers used in this study

| **Primer** | **Sequence (5'-3')** | **Template** | | **Product** |  |
| --- | --- | --- | --- | --- | --- |
| pRSF1 Lin F | taagcttaattagctgagcttggac | pRSF1 | | Linearized  pRSF1 | |
| pRSF1 Lin R | catagttaatttctcctctttaatgaattctgtgt |  |  |  |  |
| pRSF1 Lpp F | gaggagaaattaactatgaaagctactaaactggtactgggc | Synthetic  Lpp OmpA gene | | pRSF1 Lpp OmpA  insert | |
| pRSF1 Lpp R | tgaacctcctccagaacctccgccgttgtccggacgagtgcc |  |  |  |  |
| LppOmpA-Coil A F | ggttctggaggaggttcactggaaatagaggccgc | Synthetic  Coil A gene | | Coil A insert | |
| LppOmpA- Coil A R | ctcagctaattaagcttaatgatgatgatgatgatgcagcgggccgtatcttgt |  |  |  |  |
| pQE80 Lin F | tgattagctgagcttggactcctg | pQE80 | | Linearized  pQE80 | |
| pQE80 Lin R | catagttaatttctcctctttaatgaattctgtgt |  |  |  |  |
| pQE80 Lpp F | gaggagaaattaactatgaaagctactaaactggtactgggc | Synthetic  Lpp OmpA gene | | pQE80 Lpp OmpA  insert 1 | |
| pQE80 Lpp R | gctccctcctccagaacctccgccgttgtccggacgagtgcc |  |  |  |  |
| LppOmpA- Coil B F | ggttctggaggagggagcgaagtcagcgctcttgagaagg | Synthetic  Coil B gene | | Coil B insert | |
| LppOmpA- Coil B R | tccaagctcagctaatcacttgtcgtcatcgtctttgtagtctttctccaatgcagacacctcc |  |  |  |  |
| pQE80  Lpp OmpA-Coil B Lin F | ttagctgagcttggactcctgt | pQE80  Lpp OmpA-Coil B | | Linearized  pQE80  Lpp OmpA-Coil B | |
| pQE80  Lpp OmpA-Coil B Lin R | tcacttgtcgtcatcgtctttgtagtc |  |  |  |  |
| pQE80 Lpp2 F | gacgatgacgacaagtgatttcacacagaattcattaaagaggagaaatt | pQE80  Lpp OmpA-Coil B | | pQE80 Lpp OmpA  insert 2 | |
| pQE80 Lpp2 R | ggtaacaataaacgctttgctccctcctccagaacct |  |  |  |  |
| LppOmpA-Coil C F | aaagcgtttattgttaccgatgaagatattcg | Synthetic  Coil C gene | | Coil C insert | |
| LppOmpA-Coil C R | tccaagctcagctaactaagcgtaatctggaacatcgtatgggtagctcagaatatccgccatcagc |  |  |  |  |
| WT Lpp OmpA  Insert F  (pRSF1 Lpp F) | gaggagaaattaactatgaaagctactaaactggtactgggc | Synthetic  Lpp OmpA gene | WT Lpp OmpA  insert | |  |
| WT Lpp OmpA  Insert R | tccaagctcagctaatcagttgtccggacgagtgcc |  |  |  |  |
| pQE80 sfGFP  Insert F | gaggagaaattaactatgcatcaccatcaccatcacagtaaagga | Synthetic  sfGFP gene | sfGFP  insert | |  |
| pQE80 sfGFP  Insert R | tgaacccccacctcctgacc |  |  |  |  |
| sfGFP-Coil A'  Insert F | tcaggaggtgggggttcactggaaataagagcagcggcattgc | Synthetic  Coil A' gene | Coil A' insert | |  |
| sfGFP-Coil A'  Insert R | tccaagctcagctaatcacagcgggccatatctggtttcg |  |  |  |  |
| sfGFP-Coil B'  Insert F | tcaggaggtgggggttcaaaagtttccgcccttaaggaaaa | Synthetic  Coil B' gene | Coil B' insert | |  |
| sfGFP-Coil B'  Insert R | tccaagctcagctaatcattccttcagtgcgctcactt |  |  |  |  |
| sfGFP-Coil C'  Insert F | tcaggaggtgggggttcaccggaagaacgcgaacgg | Synthetic  Coil C' gene | Coil C' insert | |  |
| sfGFP-Coil C'  Insert R | tccaagctcagctaatcatttctgtgcggttgcttctttct |  |  |  |  |
| pQE80 Coil  Lin F | gggggaggcggcagt | pQE80  sfGFP-Coil | Linearized  pQE80 Coil | |  |
| pQE80 Coil  Lin R | gtgatggtgatggtgatgcatagtt |  |  |  |  |
| mCherry Insert F | catcaccatcaccatcacgtttctaaaggtgaagaagataacatggcg | Synthetic  mCherry gene | mCherry  insert | |  |
| mCherry Insert R | tccactgccgcctccccctttatacagttcatccataccaccggt |  |  |  |  |
| EBFP Insert F | catcaccatcaccatcacgtgagcaagggcgagga | Synthetic  EBFP gene | EBFP insert | |  |
| EBFP Insert R | tccactgccgcctccccccttgtacagctcgtccatgc |  |  |  |  |
| DR5 Nb3 Insert F | catcaccatcaccatcacgaagtgcagctggttgaaagc | Synthetic  DR5 Nb3 gene | DR5 Nb3 insert | |  |
| DR5 Nb3 Insert R | tccactgccgcctcccccgctagaaacggtaacctgggt |  |  |  |  |
| αEGFR Nb Insert F | catcaccatcaccatcaccaggttaaactggaagaatctggtg | Synthetic  αEGFR Nb gene | αEGFR Nb insert | |  |
| αEGFR Nb Insert R | tccactgccgcctcccccagaagaaacggtaacctgggtac |  |  |  |  |

· Underlined sequences represent overhangs.

**Table S3.** Summary of coiled-coil motifs used for surface display and payload interaction.

| **Name** | **Original**  **name** | **Origin** | **Length**  **(AA)** | **Affinity**  **(K_D_)** | **Amino acid sequence** | | | | | | | | | | | | | | | | | | | | | | | | | | | | | | | | | | | | | | | | | | | | |
| --- | --- | --- | --- | --- | --- | --- | --- | --- | --- | --- | --- | --- | --- | --- | --- | --- | --- | --- | --- | --- | --- | --- | --- | --- | --- | --- | --- | --- | --- | --- | --- | --- | --- | --- | --- | --- | --- | --- | --- | --- | --- | --- | --- | --- | --- | --- | --- | --- | --- |
|  |  |  |  |  | **d** | **e** | **f** | **g** | **a** | **b** | **c** | **d** | **e** | **f** | **g** | **a** | **b** | **c** | **d** | **e** | **f** | **g** | **a** | **b** | **c** | **d** | **e** | **f** | **g** | **a** | **b** | **c** | **d** | **e** | **f** | **g** | **a** | **b** | **c** | **d** | **e** | **f** | **g** | **a** | **b** | **c** | **d** | **e** | **f** |
| **Coil A** | ZE | Human | 43 | 1 fM^[1]^ | L | E | I | E | A | A | A | L | E | Q | E | N | T | A | L | E | T | E | V | A | E | L | E | Q | E | V | Q | R | L | E | N | I | V | S | Q | Y | R | T | R | Y | G | P | L |  |  |
| **Coil A'** | ZR | Human | 43 |  | L | E | I | R | A | A | A | L | R | R | R | N | T | A | L | R | T | R | V | A | E | L | R | Q | R | V | Q | R | L | R | N | E | V | S | Q | Y | E | T | R | Y | G | P | L |  |  |
| **Coil B** | E5 | De novo | 35 | 63 pM^[2]^ |  |  |  | E | V | S | A | L | E | K | E | V | S | A | L | E | K | E | V | S | A | L | E | K | E | V | S | A | L | E | K | E | V | S | A | L | E | K |  |  |  |  |  |  |  |
| **Coil B'** | K5 | De novo | 35 |  |  |  |  | K | V | S | A | L | K | E | K | V | S | A | L | K | E | K | V | S | A | L | K | E | K | V | S | A | L | K | E | K | V | S | A | L | K | E |  |  |  |  |  |  |  |
| **Coil C** | MBD2 | Human | 34 | 12.4 nM^[3]^ |  |  | K | A | F | I | V | T | D | E | D | I | R | K | Q | E | E | R | V | Q | Q | V | R | K | K | L | E | E | A | L | M | A | D | I | L | S |  |  |  |  |  |  |  |  |  |
| **Coil C'** | p66α | Human | 41 |  |  |  |  |  | P | E | E | R | E | R | M | I | K | Q | L | K | E | E | L | R | L | E | E | A | K | L | V | L | L | K | K | L | R | Q | S | Q | I | Q | K | E | A | T | A | Q | K |

* Key contact residues were highlighted: Hydrophobic residues; Ionic/polar residues; Asparagine lock.

**Table S4.** Flow cytometry data of gated TriSCs and WT cells treated with sfGFP-Coil payloads.

|  | **TriSC only** | | **WT only** | | **TriSC +**  **sfGFP**–**Coil A'** | | **WT +**  **sfGFP**–**Coil A'** | | **TriSC +**  **sfGFP**–**Coil B'** | | **WT +**  **sfGFP-Coil B'** | | **TriSC +**  **sfGFP**–**Coil C'** | | **WT +**  **sfGFP**–**Coil C'** | |
| --- | --- | --- | --- | --- | --- | --- | --- | --- | --- | --- | --- | --- | --- | --- | --- | --- |
| **Events** | | 9,961 | | 9,820 | | 9,666 | | 9,857 | | 9,932 | | 9,994 | | 9,688 | | 9,996 |
| **% Total** | | 99.61 | | 98.20 | | 96.66 | | 98.57 | | 99.32 | | 99.94 | | 96.88 | | 99.96 |
| **FSC-A Mean** | | 23,916.5 | | 25,545.1 | | 46,096.1 | | 24,718.5 | | 24,468.5 | | 16,654.4 | | 26,148.2 | | 18,832.4 |
| **SSC-A Mean** | | 29,345.7 | | 25,561.7 | | 37,845.5 | | 26,558.4 | | 33,689.2 | | 21,749.7 | | 32,015.9 | | 24,760.4 |
| **GFP-H Mean** | | 19.2 | | 15.6 | | 38195.3 | | 267.6 | | 3839.2 | | 42.5 | | 1,528.1 | | 37.0 |

**Table S5**. Flow cytometry analysis of TriSCs and WT cells.

**(A) EBFP Channel (Ex 405 nm, Em 450/50 nm)**

| **Condition** | **Raw MFI ± SD** | ^a)^**Corrected MFI ± Propagated SD** |
| --- | --- | --- |
| TriSC only | 25.32 ± 12.82 | - |
| TriSC + sfGFP–Coil A′ (SL) | 435.16 ± 319.85 | 409.84 ± 320.10 |
| TriSC + mCherry–Coil B′ (SL) | 53.37 ± 203.69 | 28.05 ± 204.09 |
| TriSC + EBFP–Coil C′ (SL) | 1563.28 ± 1152.24 | 1537.96 ± 1152.31 |
| TriSC + Triple (ML) | 723.99 ± 426.61 | 698.67 ± 426.80 |
| ^b)^**Retention (EBFP, ML vs SL): 45.4%** | | |

**(B) sfGFP Channel (Ex 488 nm, Em 530/30 nm)**

| **Condition** | **Raw MFI ± SD** | **Corrected MFI ± Propagated SD** |
| --- | --- | --- |
| TriSC only | 19.22 ± 53.59 | - |
| TriSC + sfGFP–Coil A′ (SL) | 26964.01 ± 20116.00 | 26944.79 ± 20116.07 |
| TriSC + mCherry–Coil B′ (SL) | 248.91 ± 2439.78 | 229.69 ± 2440.37 |
| TriSC + EBFP–Coil C′ (SL) | 123.52 ± 434.84 | 104.30 ± 438.14 |
| TriSC + Triple (ML) | 17108.34 ± 13325.67 | 17089.12 ± 13325.78 |
| **Retention (sfGFP, ML vs SL): 63.4%** | | |

**(C) mCherry Channel (Ex 488 nm, Em 700/54 nm)**

| **Condition** | **Raw MFI ± SD** | **Corrected MFI ± Propagated SD** |
| --- | --- | --- |
| TriSC only | 14.43 ± 6.30 | - |
| TriSC + sfGFP–Coil A′ (SL) | 119.01 ± 96.60 | 104.58 ± 96.82 |
| TriSC + mCherry–Coil B′ (SL) | 236.58 ± 174.07 | 222.15 ± 174.18 |
| TriSC + EBFP–Coil C′ (SL) | 15.20 ± 7.10 | 0.77 ± 9.49 |
| TriSC + Triple (ML) | 506.20 ± 348.51 | 491.77 ± 348.57 |
| **Retention (mCherry, ML vs SL):** ^c)^**n/a** | | |

**(D) EBFP Channel (Ex 405 nm, Em 450/50 nm)**

| **Condition** | **Raw MFI ± SD** | **Corrected MFI ± Propagated SD** |
| --- | --- | --- |
| WT only | 25.10 ± 36.25 | - |
| WT + sfGFP–Coil A′ (SL) | 26.19 ± 16.32 | 1.09 ± 39.70 |
| WT + mCherry–Coil B′ (SL) | 26.08 ± 11.65 | 0.98 ± 38.09 |
| WT + EBFP–Coil C′ (SL) | 64.12 ± 73.28 | 39.02 ± 81.77 |
| WT + Triple (ML) | 61.99 ± 51.08 | 36.89 ± 62.69 |

**(E) sfGFP Channel (Ex 488 nm, Em 530/30 nm)**

| **Condition** | **Raw MFI ± SD** | **Corrected MFI ± Propagated SD** |
| --- | --- | --- |
| WT only | 15.62 ± 6.46 | - |
| WT + sfGFP–Coil A′ | 170.74 ± 1135.36 | 155.12 ± 1135.38 |
| WT + mCherry–Coil B′ | 15.80 ± 6.50 | 0.18 ± 9.17 |
| WT + EBFP–Coil C′ | 15.83 ± 7.47 | 0.21 ± 9.85 |
| WT + Triple (ML) | 76.88 ± 216.68 | 61.26 ± 216.78 |

**(F) mCherry Channel (Ex 488 nm, Em 700/54 nm)**

| **Condition** | **Raw MFI ± SD** | **Corrected MFI ± Propagated SD** |
| --- | --- | --- |
| WT only | 14.40 ± 7.24 | - |
| WT + sfGFP–Coil A′ | 16.51 ± 11.30 | 2.11 ± 13.40 |
| WT + mCherry–Coil B′ | 17.89 ± 11.08 | 3.49 ± 13.24 |
| WT + EBFP–Coil C′ | 14.43 ± 6.46 | 0.03 ± 9.71 |
| WT + Triple (ML) | 19.38 ± 11.20 | 4.98 ± 13.40 |

^a)^ Baseline correction was performed by subtracting the Cell-only control. Propagated SD was calculated as$\sqrt{{SD}_{sample}^{2}+ {SD}_{Cell-only}^{2}}$.

^b)^ Retention (%) was calculated as (Corrected ML / Corrected SL) × 100.

^c)^ Quantitative interpretation of mCherry retention was not performed due to excitation mismatch (488 nm) and substantial sfGFP-derived cross-excitation under this optical configuration.

**Figure S1.** Heptad repeat diagrams of Coil A-Coil A' (A), Coil B-Coil B' (B), and Coil C-Coil C' (C). Key contact residues were highlighted: Hydrophobic residues; Ionic/polar residues; Asparagine lock**.**

**
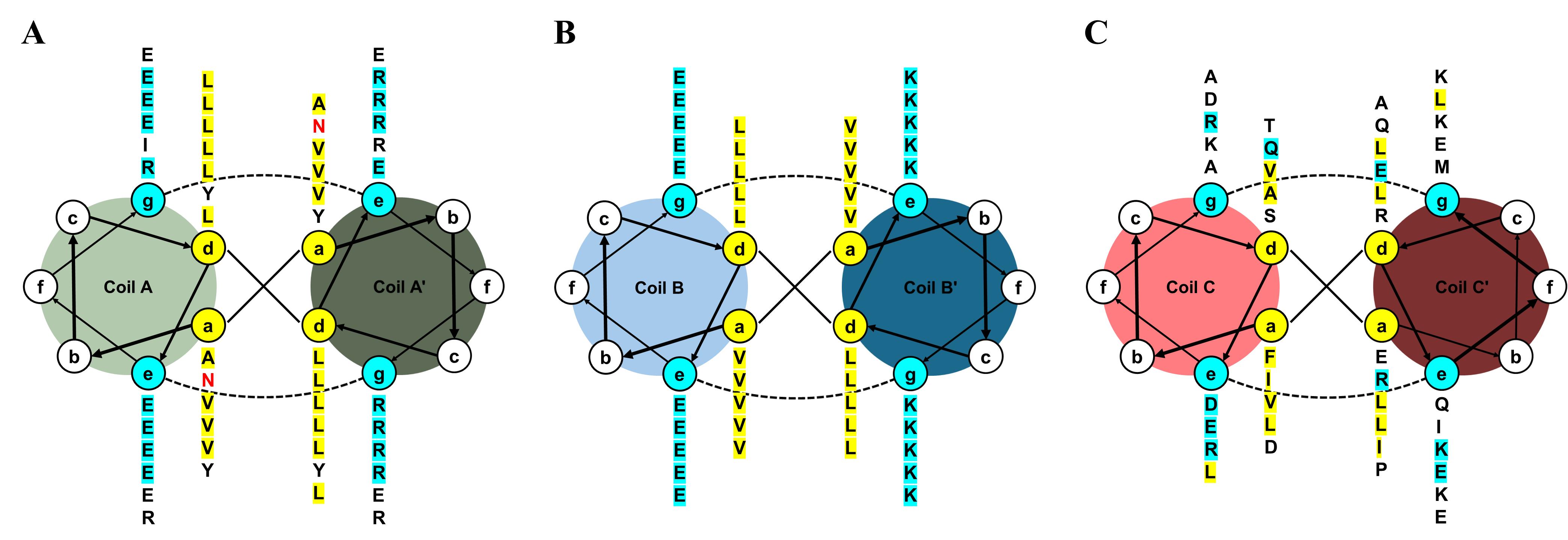
**

**
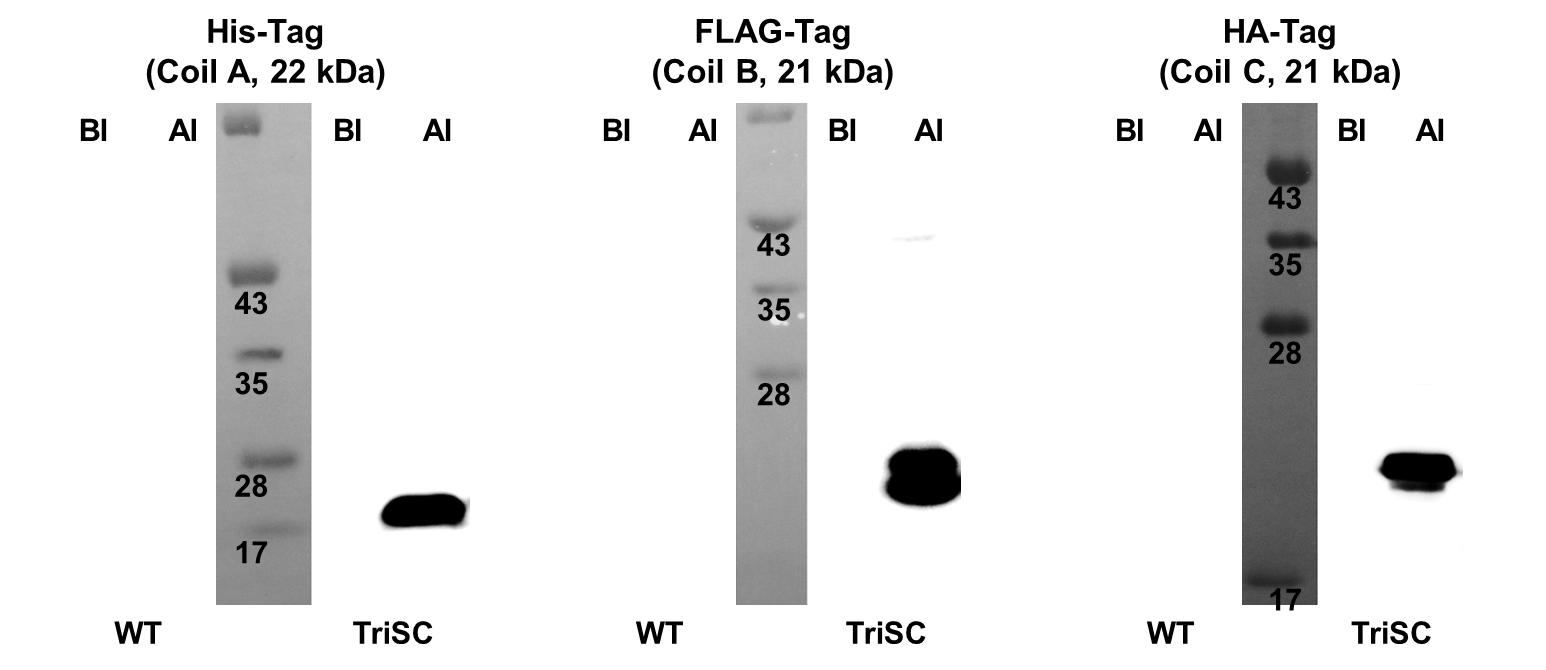
**

**Figure S2.** Images of the uncropped Western blots shown in Figure 2C

**
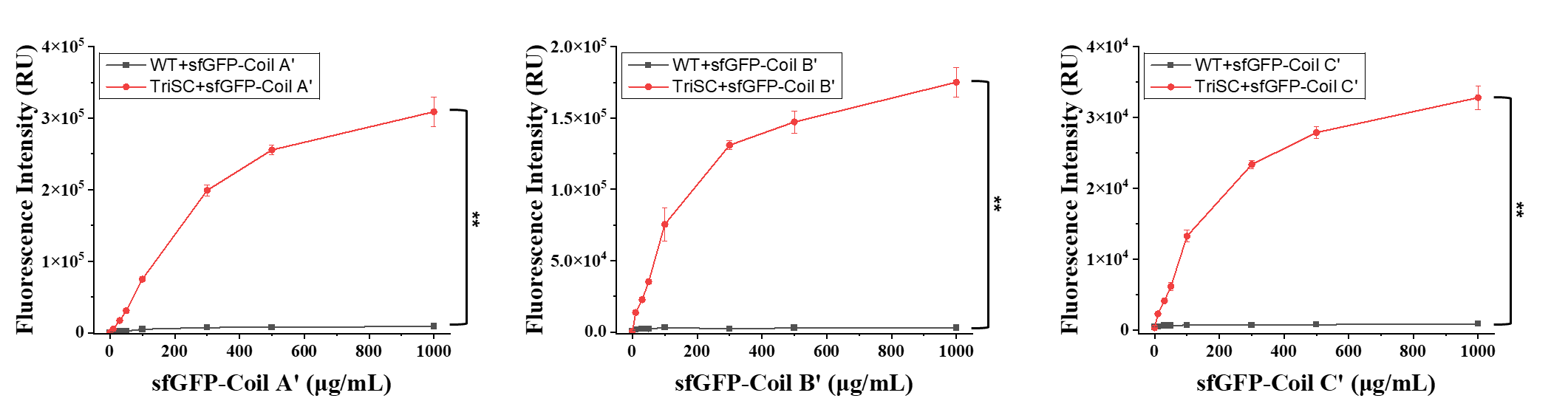
**

**Figure S3.** Quantitative fluorescence analyses of TriSCs and WT cells after incubation with sfGFP–Coil A', sfGFP–Coil B', or sfGFP–Coil C'. Fluorescence signals were measured using a microplate reader (Excitation 485 nm / Emission 520 nm). All data are means ± standard deviation (SD) with n = 3 (biological replicates). Statistical significance was determined by Welch's t-test (**P <* 0.05; ***P <* 0.01; ****P <* 0.001; ns, *P* > 0.05).

**
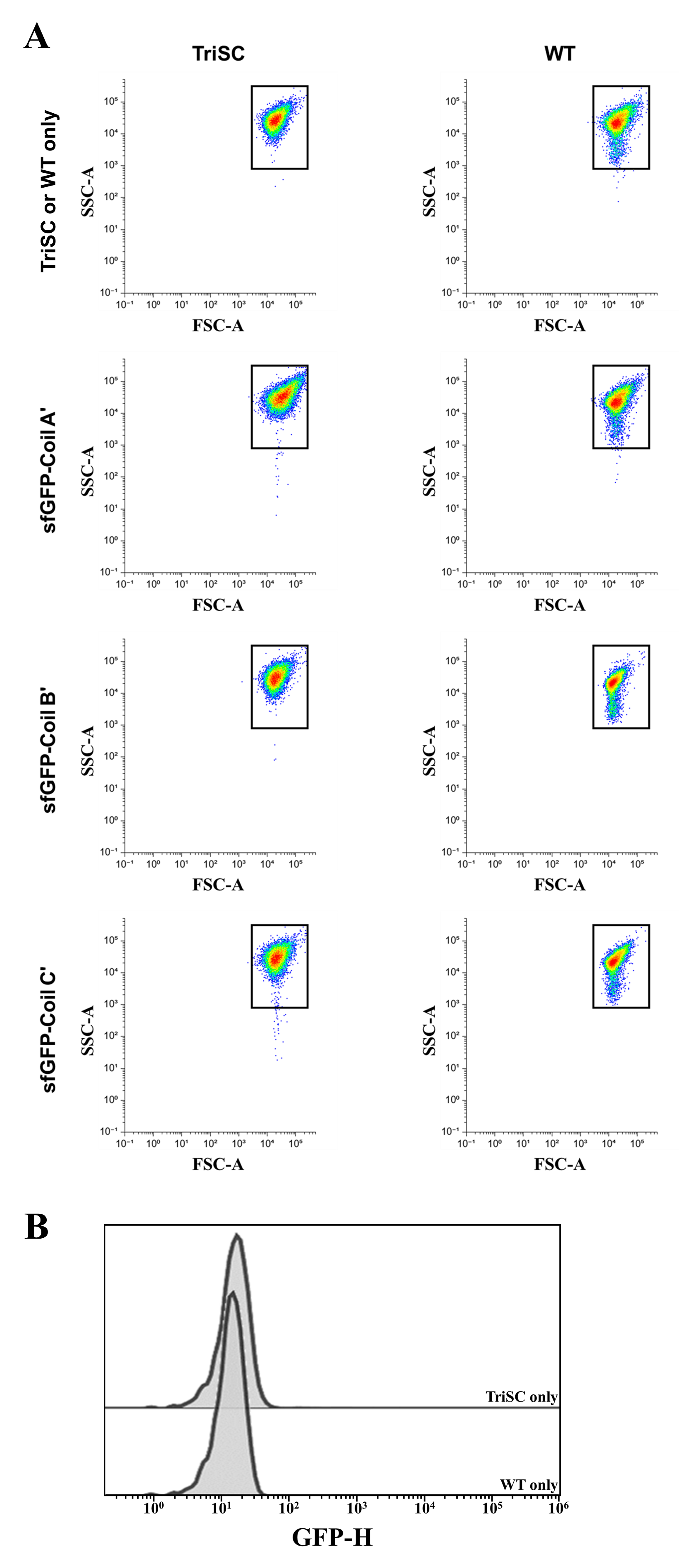
**

**Figure S4.** Flow cytometry analysis of TriSCs and WT cells. A) FSC-A and SSC-A plots of TriSC and WT cells under various conditions. B) Fluorescence histograms of TriSC and WT cells without any payloads.

**
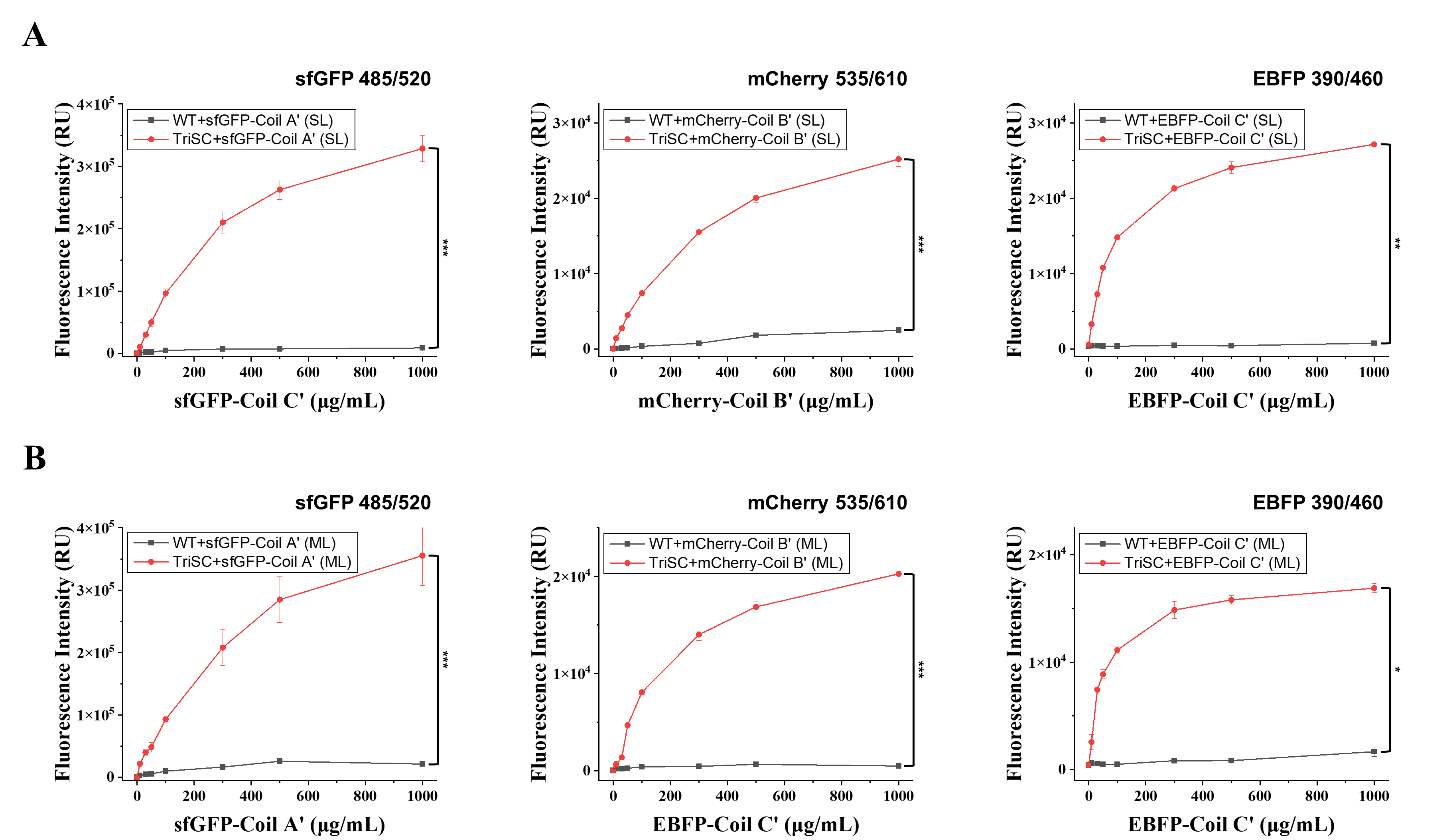
Figure S5.** Quantitative fluorescence analysis of TriSCs and WT cells after A) singleplex loading or B) multiplex loading of fluorescent payloads. Fluorescence signals were quantified using a microplate reader at fluorophore-specific excitation/emission settings: 390/460 nm for EBFP, 485/520 nm for sfGFP, and 535/610 nm for mCherry. All data shown on A) and B) are means ± SD (n = 3, biological replicates). Statistical significance was determined by Welch's t-test (**P <* 0.05; ***P <* 0.01; ****P <* 0.001; ns, *P* > 0.05).


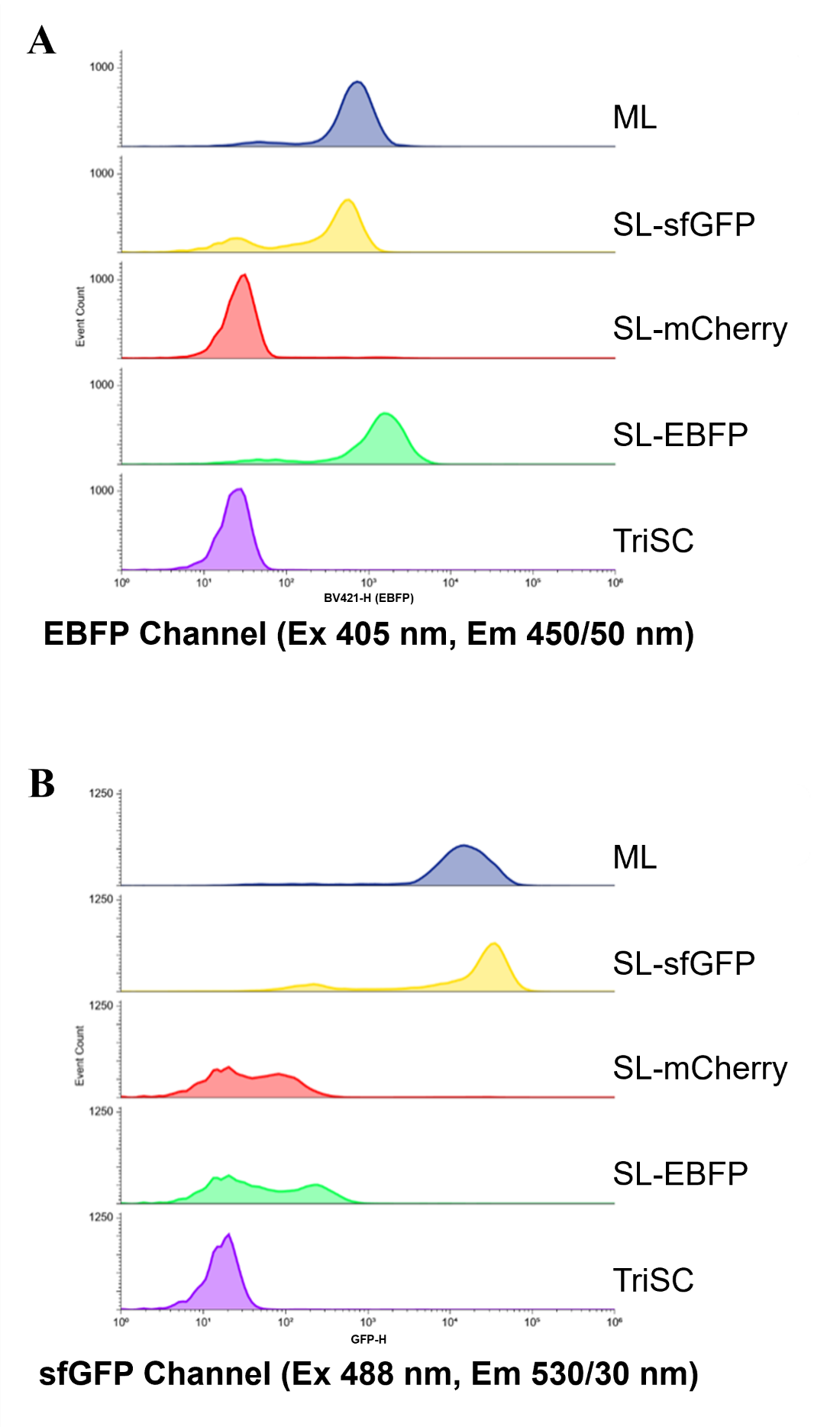


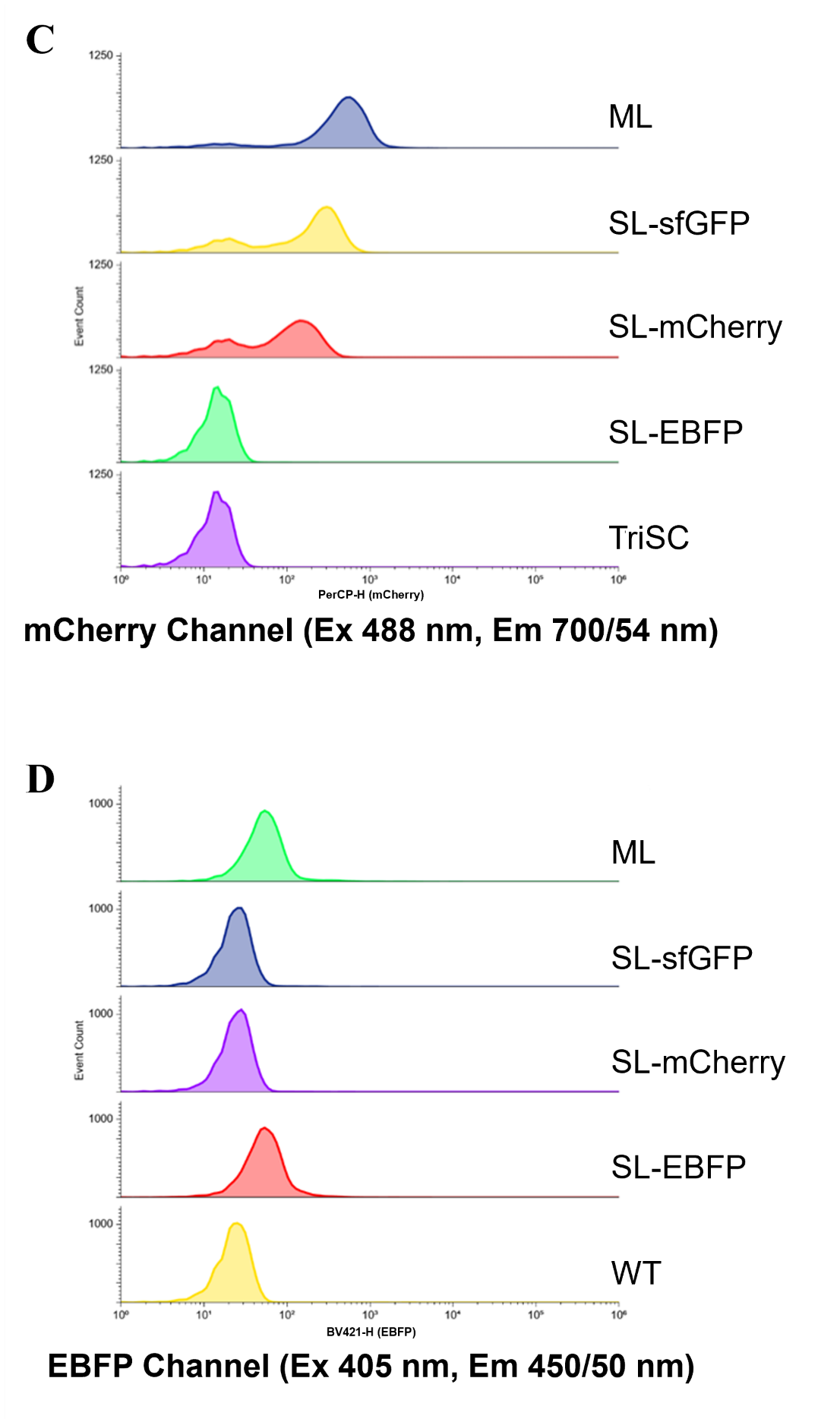


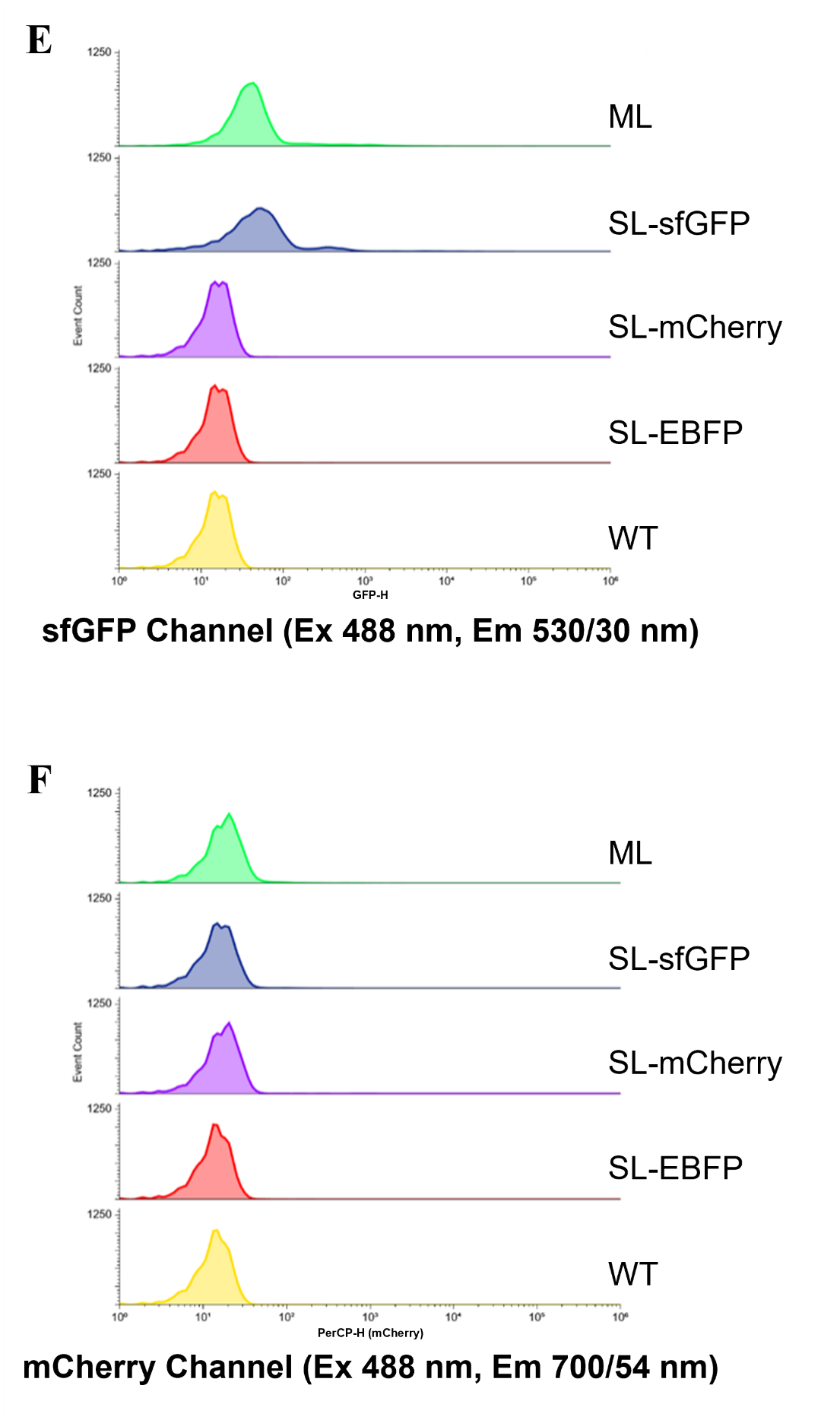


**Figure S6.** Single-cell flow cytometric analysis of singleplex (SL) and multiplex (ML) payload recruitment in TriSC and WT cells. (A–C) Flow cytometry histograms of TriSCs. (D–F) Corresponding histograms of WT cells (negative control). Fluorescence was analyzed using the following optical configurations: (A, D) EBFP channel (405 nm excitation, 450/50 nm emission); (B, E) sfGFP channel (488 nm excitation, 530/30 nm emission); (C, F) mCherry channel (488 nm excitation, 700/54 nm emission). TriSCs or WT cells were incubated with fluorescent payloads under singleplex (SL) or multiplex (ML) conditions at 500 µg/mL, followed by washing prior to acquisition. For each sample, 10,000 gated events were collected after FSC/SSC-based bacterial gating. Condition codes are defined as follows: TriSC, TriSC only; SL-A, TriSC + sfGFP–Coil A′; SL-B, TriSC + mCherry–Coil B′; SL-C, TriSC + EBFP–Coil C′; ML, TriSC incubated simultaneously with all three payloads. WT panels (D–F) represent identical payload treatments applied to WT cells lacking surface-displayed coils, serving as negative controls for nonspecific binding and autofluorescence. In TriSC cells (A–C), clear population shifts are observed under SL conditions and are preserved under ML conditions in the EBFP and sfGFP channels (A, B), consistent with microplate-based bulk fluorescence measurements (Fig. 4C). Quantitative analysis was performed for these channels using background-corrected mean fluorescence intensity (MFI) values (Table S1). For the mCherry channel (C, F), quantitative retention analysis was not performed due to excitation mismatch. The flow cytometer utilized 488 nm excitation for red-channel detection, whereas mCherry is optimally excited near 561 nm. Under 488 nm excitation, sfGFP exhibits substantially higher effective brightness and generates detectable emission extending into the red detection channel. Single-payload controls confirmed that sfGFP produced stronger apparent red-channel signal than mCherry under these conditions. Because this cross-excitation cannot be fully resolved by compensation alone, red-channel data are presented for qualitative assessment only.


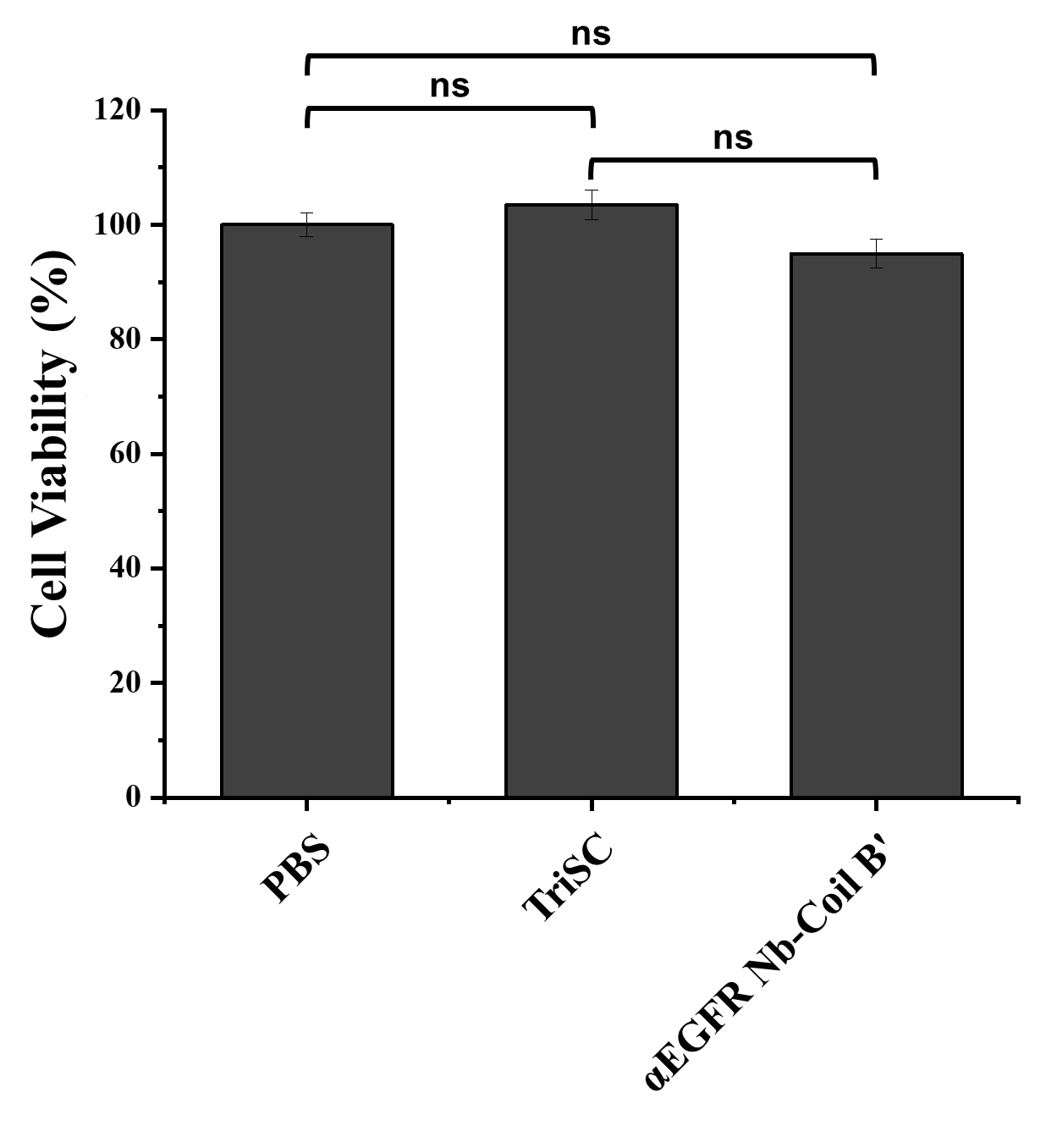


**Figure S7**. Cytotoxicity of TriSCs and nanobody treatments. Viability of Colo205 cells after treatment with TriSCs alone or with αEGFR Nb–Coil B' alone. All data are means ± SD (n = 3, biological replicates). Statistical significance was determined by Welch's t-test (**P <* 0.05; ***P <* 0.01; ****P <* 0.001; ns, *P* > 0.05).


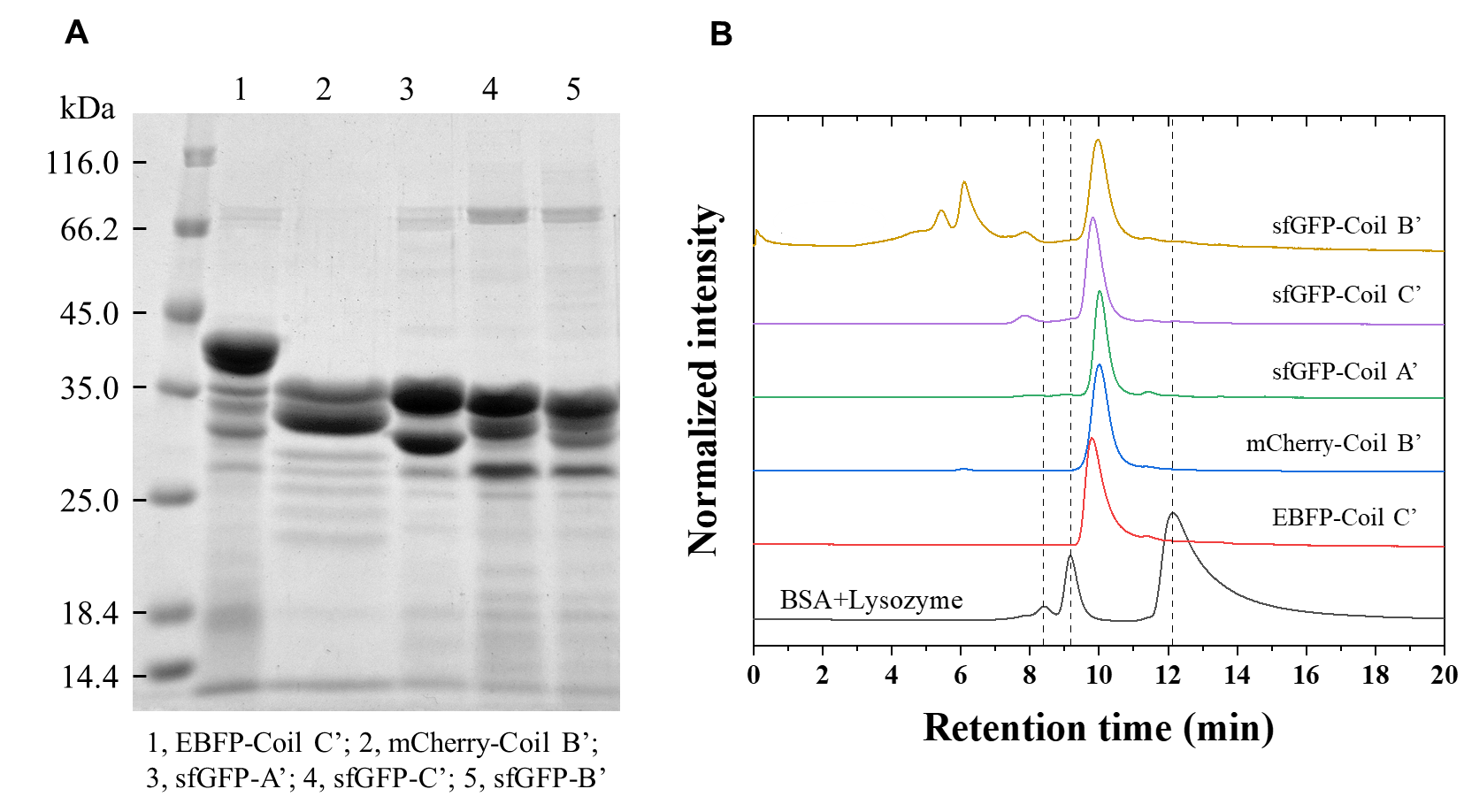


**Figure S8**. Purification and size characterization of recombinant fluorescent payload proteins. A) SDS–PAGE analysis of purified sfGFP–Coil A′, mCherry–Coil B′, and EBFP–Coil C′ following Ni-NTA affinity chromatography. The multiple bands likely reflect intrinsic structural properties of fluorescent proteins. Their highly stable β-barrel architecture can resist complete denaturation by SDS, resulting in distinct conformational states that migrate differently during electrophoresis.^[4,5]^ These banding patterns persisted even under reducing and high-temperature denaturing conditions (DTT, 95 °C), possibly due to partial refolding during electrophoresis. B) Size-exclusion chromatography (SEC) profiles obtained using a Superdex 200 Increase 10/300 GL column equilibrated in PBS (pH 7.4). Bovine serum albumin (BSA, 66 kDa) and lysozyme (14.3 kDa) were used as molecular weight standards for size calibration. All proteins eluted between BSA and lysozyme, consistent with their expected molecular weights, and as single symmetric peaks except for sfGFP–Coil B′ displaying a minor early-eluting peak at 4–6 min corresponding to a small fraction of high-molecular-weight specie**s.**

**References**

[1] J. R. Moll, S. B. Ruvinov, I. Pastan, C. Vinson, *Protein Sci.* **2001**, *10*, 649.

[2] G. De Crescenzo, J. R. Litowski, R. S. Hodges, M. D. O’Connor-McCourt, *Biochemistry* **2003**, *42*, 1754.

[3] M. N. Gnanapragasam, J. N. Scarsdale, M. L. Amaya, H. D. Webb, M. A. Desai, N. M. Walavalkar, S. Z. Wang, S. Z. Zhu, G. D. Ginder, D. C. Williams, *Proc. Natl. Acad. Sci. U. S. A.* **2011**, *108*, 7487.

[4] T. Aoki, Y. Takahashi, K. S. Koch, H. L. Leffert, H. Watabe, *FEBS Lett.* **1996**, *384*, 193.

[5] E. R. Geertsma, M. Groeneveld, D.-J. Slotboom, B. Poolman, *Proceedings of the National Academy of Sciences* **2008**, *105*, 5722.
